# ICA69 aggravates ferroptosis causing septic cardiac dysfunction via STING trafficking

**DOI:** 10.1038/s41420-022-00957-y

**Published:** 2022-04-09

**Authors:** Chang Kong, Xuqing Ni, Yixiu Wang, Anqi Zhang, Yingying Zhang, Feihong Lin, Shan Li, Ya Lv, Jingwen Zhu, Xinyu Yao, Qinxue Dai, Yunchang Mo, Junlu Wang

**Affiliations:** 1 Department of Anesthesia, The First Affiliated Hospital of Wenzhou Medical University, Wenzhou, Zhejiang China; 2 Department of General Practice, Wenzhou Central Hospital, 325000 Wenzhou, Zhejiang China

**Keywords:** Inflammation, Cell death, Cell death and immune response

## Abstract

Previous studies have demonstrated that cardiomyocyte apoptosis, ferroptosis, and inflammation participate in the progress of sepsis-induced cardiomyopathy (SIC). Although Islet cell autoantigen 69 (ICA69) is an imperative molecule that could regulate inflammation and immune response in numerous illnesses, its function in cardiovascular disease, particularly in SIC, is still elusive. We confirmed that LPS significantly enhanced the expression of ICA69 in wild-type (WT) mice, macrophages, and cardiomyocytes. The knockout of ICA69 in lipopolysaccharide(LPS)-induced mice markedly elevated survival ratio and heart function, while inhibiting cardiac muscle and serum inflammatory cytokines, reactive oxygen (ROS), and ferroptosis biomarkers. Mechanistically, increased expression of ICA69 triggered the production of STING, which further resulted in the production of intracellular lipid peroxidation, eventually triggering ferroptosis and heart injury. Intriguingly, ICA69 deficiency only reversed the ferroptotic marker levels, such as prostaglandin endoperoxide synthase 2 (PTGS2), malonaldehyde (MDA), 4-hydroxynonenal (4HNE), glutathione peroxidase 4 (GPX4), superoxide dismutase (SOD), iron and lipid ROS, but had no effects on the xCT-dependent manner. Additionally, greater ICA69 level was identified in septic patients peripheralblood mononuclear cells (PBMCs) than in normal control groups. Generally, we unveil that ICA69 deficiency can relieve inflammation and ferroptosis in LPS-induced murine hearts and macrophages, making targeting ICA69 in heart a potentially promising treatment method for SIC.

## Introduction

Sepsis is a systemic inflammatory response that is accompanied by multiple organ dysfunction, oxidative stress, and overmuch inflammatory cytokines [1] sepsis-induced cardiomyopathy (SIC) is one of the common and well-elucidated complications in sepsis and sepsis-induced shock, while Gram-negative bacterial endotoxin (Lipopolysaccharide, LPS) serves as a key sepsis mediator for septicemia-associated multiple organ dysfunction or mortality [2].

Encoded by the ICA1 gene, ICA69 has a limited cellular distribution and tissue distribution. Past study primarily highlighted the physiopathological function of ICA69 in organ-specific autoimmune illnesses including Type 1 diabetes (T1D) [3]. Thymic deletion of ICA69 expression, for instance, is adequate to give rise to inflammatory events in various organs [4]. Nevertheless, our research revealed that ICA69 was notably regulated upward in lipopolysaccharide-induced cardiac tissue. According to previous research findings, ICA69 enrichment occurs in the proximity of the Golgi complex and its N-terminal half involves a BAR domain, a component that could bend or bind membranes and has mutual effect with phosphatide [5]. And the BAR-domain family encompasses numerous constituents, the majority of which function in transport and endocytosis [6].

STING, composed of multiple assumed transmembrane regions, is primarily anchored as a homodimer in the ER membrane in resting conditions [7]. Recent work suggests that, after STING binds cGAMP, it transfers to the ER-Golgi intermedium compartment (ERGIC) and the Golgi via the process depending on the COPI (coat protein complex I), COPII complex and ARF GTPases [8, 9], which is essential for the phosphorylation of STING and subsequent IRF3 stimulation [10, 11]. As STING marks an imperative molecule that can modulate inflammation and defense response in SIC, and participates in septic heart damage via inducing cardiac muscle cell pyroptosis [12], these results hint that ICA69 may participate in STING-dependent innate immune response.

Ferroptosis is a ROS-related and iron-related cell death, which is crucial for organ damage and target treatment of tumors [13, 14]. Recent studies show that the ferroptosis induction via high-iron diet or Gpx4 consumption stimulates the STING-related DNA sensor pathway, which finally causes the infiltration of macrophagus and pancreatic tumorigenesis [15]. By comparison, the genetic inhibition of STING activation prevents ferroptosis in vitro and in vivo [16], while STING shortage mitigated inflammatory activities and oxidative stress in LPS-challenged mouse lungs and macrophagus [17]. Although we know that the cardiomyocyte ferroptosis was stimulated in mice with LPS-caused septic cardiac injury and the ferroptosis suppression could mitigate cardiac inflammatory activities and lesion [13], there remains no valid data about the function of ICA69 and STING in ferroptosis-caused heart damage.

As on the level of molecules and cells, inflammatory process, apoptosis, and ferroptosis are considered pivotal pathophysiological events in sepsis and SIC [13, 18]. Molecules or genes that target the inhibition of these activities will be rather useful for treatment of sepsis and lipopolysaccharide-induced cardiac dysfunction. Hence, our team aimed at investigating if ICA69 could impact LPS-caused heart disfunction and damage while revealing the underlying mechanisms of ICA69 in SIC.

## Results

### LPS increased ICA69 in vivo while in vitro

**Fig. 1:**
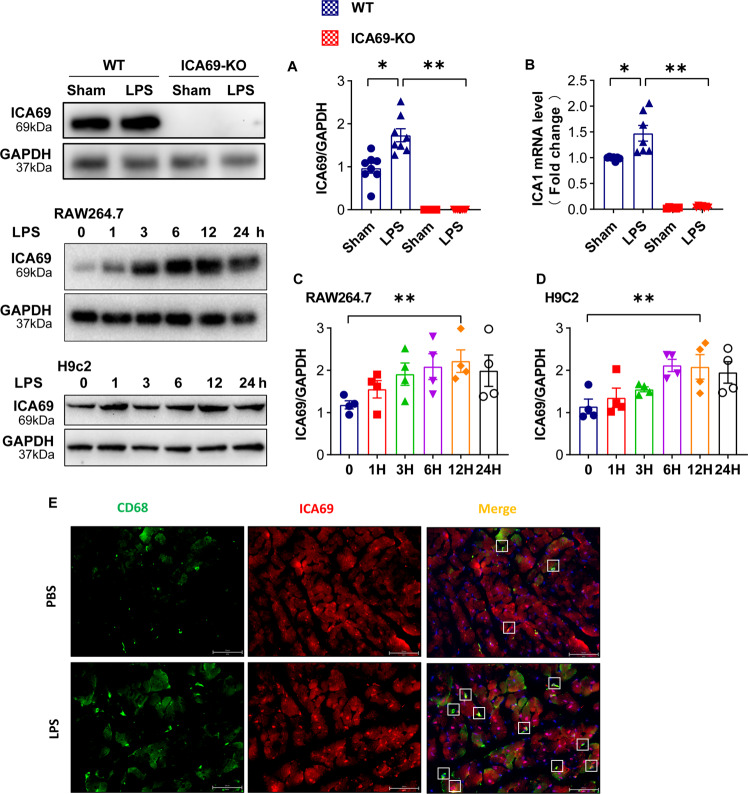
LPS increased ICA69 in vivo while in vitro. **A** The protein levels of ICA69 in mouse hearts stimulated by LPS (10 mg/kg) or PBS for 12 h. Levels of ICA69 intensity were normalized to the corresponding GAPDH level, and the changes are shown in the histograms (*n* = 8). **B** qRT-PCR was performed to detect the mRNA expression of ICA1 stimulated with LPS (10 mg/kg) or PBS for 12 h (*n* = 7). *P < 0.05 vs. LPS group, **P < 0.001 vs. ICA69-KO + LPS group. **C**, **D** Alterations of ICA69 levels in RAW264.7 cells and H9C2 myofibroblasts after LPS (1 μg/mL for 0 h, 1 h, 3 h, 6 h, 12 h, 24 h) treatment. Representative immunoblots and densitometric analysis of ICA69 in cells. Levels of ICA69 intensity were normalized to the corresponding GAPDH level, and the changes are shown in the histograms (*n* = 4 per group. **P < 0.05 vs. 0 h group). The data are expressed as the mean ± standard error of the mean. **E** Immunofluorescence staining of CD68 (green) and ICA69 (red) in the hearts of PBS or LPS-injected mice. Yellow indicates colocalization of ICA69 in macrophages (merge; white frame; scale bar = 50 μm).

To assess the underlying function of ICA69 in sepsis-induced heart dysfunction, our team studied the levels of ICA69 and the infiltration of macrophages in LPS heart lesion. Firstly, our team carried out quantitative real-time PCR and western blot observation to calculate the ICA69 level in healthy cardiac samples and those from LPS mice (10 mg/kg). Twelve hours after LPS challenge, the protein expression level and the level of ICA69 mRNA in the LPS group increased notably in comparison with that in the sham group (Fig. 1A, B). It is well-known that lipopolysaccharide (LPS) is an effective activator of sepsis to release a good amount of pro-inflammatory cytokines in monocytes [19]. Macrophages are a group of mononuclear phagocytes that participate in immune defense against invasive pathogens in heart, lung, and kidney infections [20]. RAW264.7 cells incubated by LPS is a widely accepted in vitro model for discovering sepsis [21]. Subsequently, We evaluated the level of ICA69 in RAW264.7 cells and H9c2 myofibroblasts cells challenged with LPS (1 μg/mL for 0 h, 1 h, 3 h, 6 h, 12 h, 24 h) treatment. The outcomes exhibited that the ICA69 protein level in RAW264.7 cells at 12 h was notably rised, but it did not alter at 24 h (Fig. 1C). As the level of ICA69 in H9c2 myofibroblasts showed a similar distribution pattern (Fig. 1D), the time of LPS challenge in mice and cells in following assays was 12 h.

CD68 is a commonly used marker for macrophages, immunofluorescence staining revealed that ICA69 and CD68 were colocalized, and the expression of ICA69 and CD68 were significantly increased in the LPS-induced failing hearts (Fig. 1E). As far as we know, the clinical manifestations of sepsis are in large part attributable to LPS-induced signal transduction and gene expression in both myeloid cells (eg, macrophages) and nonmyeloid cells (eg, endothelial cells and cardiomyocytes). These events are the primary cause of myocardial dysfunction in sepsis, which is an important determinant of patient outcome [22]. We predicted that ICA69 might engage in the SIC progress via both macrophages and cardiomyocytes.

### ICA69 deficiency improved the survival and cardiac function of LPS-treated mice and suppressed cardiac inflammation

**Fig. 2:**
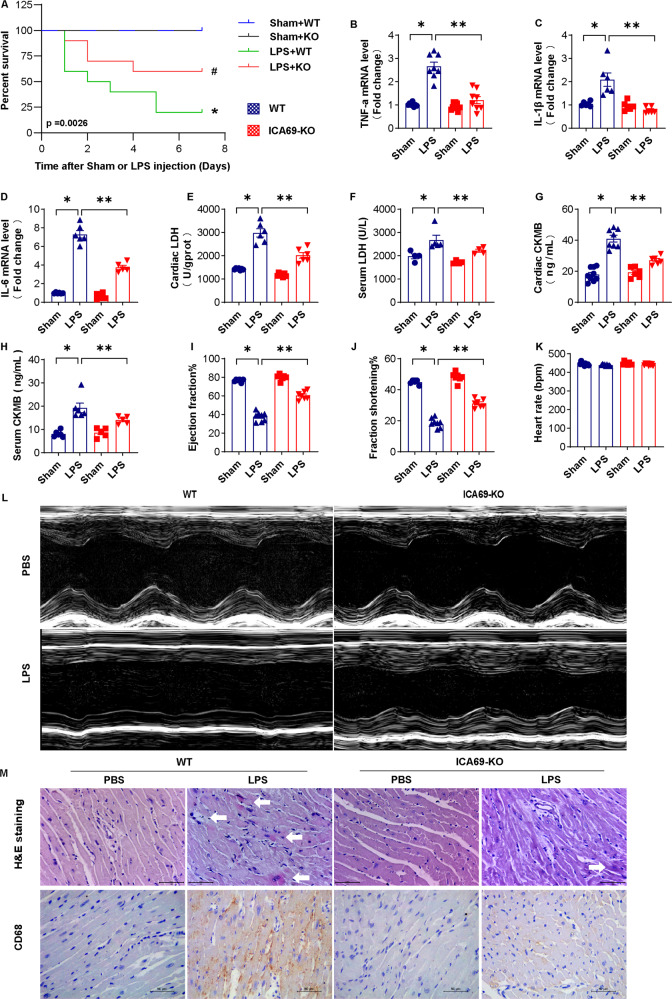
ICA69 deficiency improved the survival and cardiac function of LPS-treated mice and suppressed cardiac inflammation. **A** Survival curves of ICA69-deficient (KO) and wild-type(WT) mice after LPS challenge. Male ICA69 KO and WT mice at 8 weeks of age were stimulated with LPS or PBS, respectively. The mice were observed for their health status a few times per day for 1 week. Data were analyzed by Kaplan–Meier log-rank test. *n* = 10 per group. *P < 0.05 vs. Sham + WT group, #*P* < 0.05 vs. LPS + WT group. **B**–**D** The mRNA levels of TNF-α, IL-1β, and IL-6 in myocardial tissues of each group (*n* = 8/6/6). **E**, **F** Effect of ICA69 deficiency on LPS-induced LDH release in cardiac tissue and serum (*n* = 6/5). **G**, **H** Effect of ICA69 deficiency on LPS-induced CK-MB release in cardiac tissue and serum (*n* = 7/5). **I**–**L** Effect of ICA69 deficiency on left ventricle ejection fraction, left ventricle fractional shortening, and heart rate of each group of each group after Sham or LPS injection (*n* = 7). The data are expressed as the mean ± standard error of the mean. *P < 0.05 vs. LPS group, **P < 0.05 vs. ICA69-KO + LPS group. **M** Representative images of the morphological analysis and inflammatory cells infiltration as reflected by the H&E staining, and immunohistochemistry staining for CD68 protein. The arrow marks inflammatory cells (*n* = 3, 10 + fields per heart).

Afterwards, our team studied the role of ICA69 in survival, heart damage, and heart function by intraperitoneally injecting maleICA69-KO and WT(wild-type) mice (8 weeks old) with either LPS or an equal volume of PBS. As presented in Fig. 2A, our team computed the survival ratio in seven days in the four groups below: Sham + WT, Sham + ICA69 KO, LPS + WT, and LPS + ICA69 KO. The survival ratio in seven days in Sham + WT group and Sham + ICA69 KO group were nearly a hundred percent, whereas 7 days posterior to LPS treatment, the survival rate of LPS + WT group dropped notably to 20%. With the ICA69 knockout, the survival ratio in LPS + ICA69 KO group rised to 60%. The activation of inflammatory response marks one of the most essential pathology variations in sepsis-caused cardiac muscle injury, whereas bacterial dsDNA, along with endotoxin, could trigger both type I IFN production and other proinflammatory cytokines [18]. Hence, our team studied inflammatory cell infiltration and proinflammatory cytokines mRNA expression in all groups. As presented in Fig. 2B–D, the mRNA levels of TNF-α, IL-1β, and IL-6 rised dramatically in LPS + WT group relative to Sham + WT group, whereas the ICA69 shortage diminished the pro-inflammatory cytokines levels. As LDH and CK-MB release are sensitive biomarkers of heart damage, our team measured the LDH level and CK-MB level in the myocardial tissue and serum (Fig. 2E–H), discovering that ICA69 shortage notably decreased the LDH level and CK-MB level in mice injected with LPS. By echocardiography, our research studied as well the heart function in four groups and found ICA69 deficiency reinforced as well the fraction shortening% and ejection fraction% in mice exposed to LPS (Fig. 2I–L). The myocardium slices were dyed by H&E to evaluate the heart muscle injury and inflammatory cell infiltration (Fig. 2M), the latter of which was notably presented by the photomicrographic sections of LPS + WT group. However, in LPS + ICA69 KO group, the myocardium fibers registered obvious striation and little inflammatory infiltration was detected in heart muscle tissues. In line with histological staining, Immumohistochemical staining further disclosed that the infiltration of CD68-labeled macrophages caused by LPS was inhibited in LPS + ICA69 KO group. In summary, those outcomes exhibited that ICA69 shortage might induce cardioprotection via inhibiting heart inflammatory events in LPS mice.

### ICA69 affected the expression level of STING

**Fig. 3:**
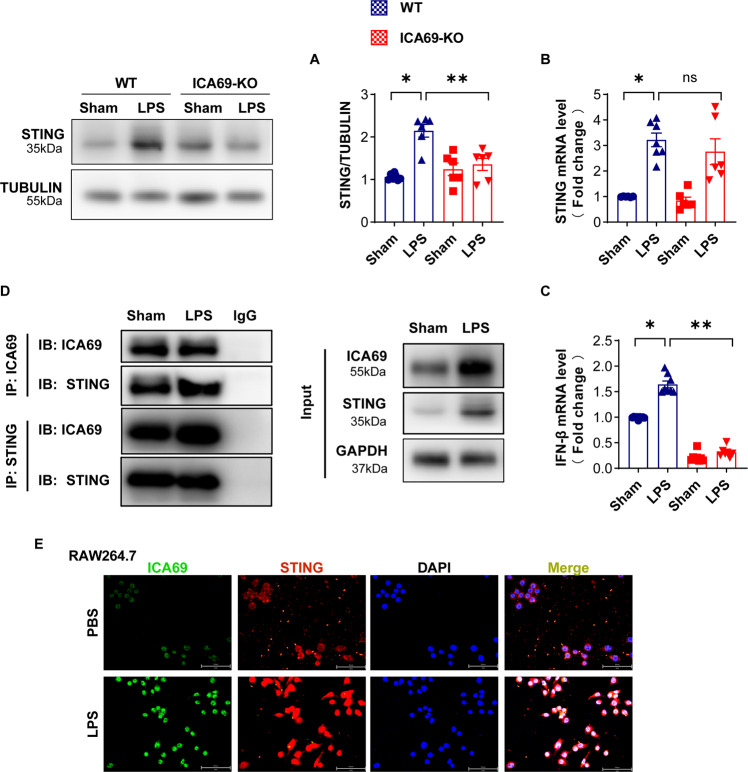
ICA69 affected the expression level of STING. **A** Representative immunoblots and densitometric analysis of total STING in cardiac tissue of ICA69-deficient (KO) and wild-type (WT) mice after LPS challenge. Levels of total STING intensity were normalized to the corresponding TUBULIN level, and the changes are shown in the histograms (*n* = 6). **B**, **C** The mRNA levels of STING and IFN-β in indicated groups that were stimulated with LPS or PBS for 12 h (*n* = 6). The data are expressed as the mean ± standard error of the mean.*P < 0.05 vs. LPS group, **P < 0.05 vs. ICA69-KO + LPS group, ns no significance. **D** The effect of LPS for the alterations of ICA69 and STING interaction in cardiac tissues. Representative western blot bands of ICA69 and STING in cardiac tissue by co-IP (*n* = 3 biologically independent experiments). **E** Representative images of immunofluorescence of ICA69 and STING in RAW264.7 cells stimulated by LPS (1 μg/mL) for 12 h. (scale bar = 50 μm).

To identify whether ICA69 deficiency impairs STING signaling, we treated mice with lipopolysaccharide. Twelve hours after LPS (10 mg/kg) injection, western blot confirmed that ICA69-/- mice with LPS significantly produced less STING in the cardiac tissues compared with those in the tissues of wild-type controls injected with LPS (Fig. 3A). Interestingly, the mRNA level of STING in LPS + WT was significantly increased compared with that in Sham + WT group, however there was no statistical significance between LPS + WT and LPS + ICA69 KO (Fig. 3B). Those outcomes exhibited that ICA69 affected STING at protein level but not mRNA. Furthermore, STING can be activated by LPS, triggering the activation of inherent immune responses including the level of the cytokines type I interferon (IFNs) and interleukins [7, 23]. We observed that ICA69 deficiency significantly reversed the increased mRNA level of IFN-β in LPS-treated mice as expected (Fig. 3C). To further explore the relationship of ICA69 and STING in LPS-treated murine cardiac tissue, we performed CO-IP assays, which showed that LPS increased STING level that co-immunoprecipitated with ICA69. LPS also triggered increased complex of ICA69 that co-immunoprecipitated with STING (Fig. 3D). Likewise, our research discovered notable colocalization between ICA69 and STING in RAW264.7 after LPS stimulation, and the expression of ICA69 and STING were significantly increased (Fig. 3E). These results indicate that ICA69, as an affirmative mediator of STING signaling and, can directly combind to STING. We suspect that ICA69 may further promote the transport of STING from endoplasmic reticulum to Golgi.

### Verification of ferroptosis markers in ICA69−/− mice with sepsis-induced cardiac injury

Previous studies have demonstrated that LPS resulted in a significant rise of ROS, the expression of 4-hydroxynonenal (4-HNE) and malonaldehyde (MDA) content, and a markedly decline of SOD process, which was practically reversed by STING shortage [17]. Hence, we explored whether the variation of ICA69 participates in ferroptosis in septic cardiomyopathy. Then, our team researched in depth the level of ferroptosis biomarkers such as cyclooxygenase-2 (COX-2) and glutathione peroxidase 4 (GPX4) in mice with sepsis-induced cardiomyopathy. COX-2, named as prostaglandin endoperoxide synthase 2 (PTGS2) as well, is an identified biomarker of ferroptosis [24], the elevated level of which was detected as well in septic mouse hearts [13, 25]. The lipid hydroperoxidase GPX4 serves as an essential upstream ferroptosis mediator [26, 27].

**Fig. 4:**
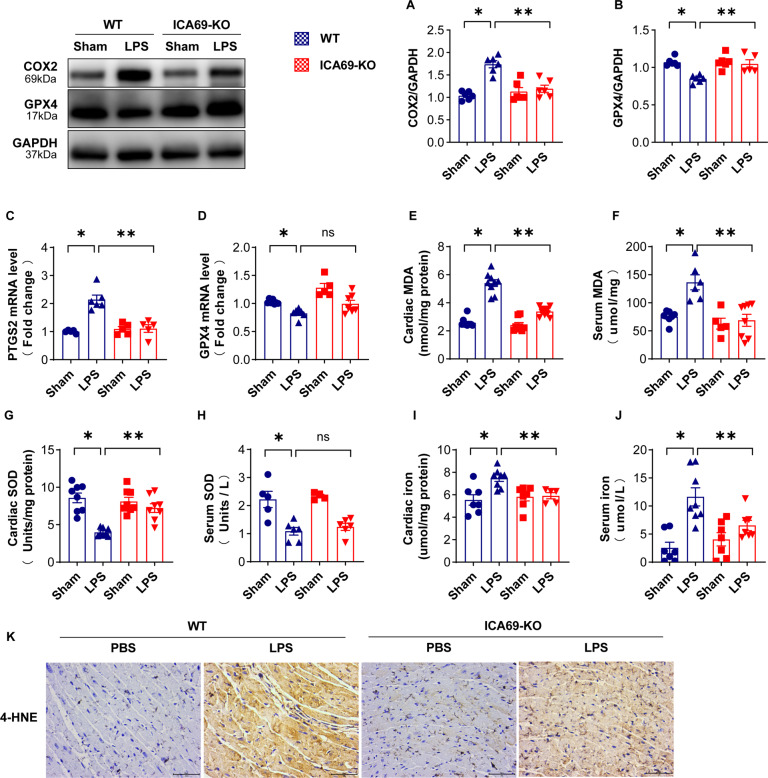
Verification of ferroptosis markers in ICA69-/- mice with sepsis-induced cardiac injury. **A**, **B** Representative immunoblots and densitometric analysis of total COX2 and GPX4 in cardiac tissue of ICA69-deficient (KO) and wild-type (WT) mice after LPS challenge. Levels of total COX2 and GPX4 intensity were normalized to the corresponding GAPDH level, and the changes are shown in the histograms (*n* = 6). **C**, **D** The mRNA levels of PTGS2 and GPX4 in the indicated groups (*n* = 6). **E**, **F** The level of MDA in the indicated groups in cardiac tissue and serum (*n* = 8/6). **G**, **H** The level of SOD in the indicated groups in cardiac tissue and serum (*n* = 8/6). **I**, **J** The level of iron in the indicated groups in cardiac tissue and serum (*n* = 6/7). The data are expressed as the mean ± standard error of the mean. *P < 0.05 vs. WT + LPS group, **P < 0.05 vs. KO + LPS group, ns no significance. **K** Immunohistochemistry staining for 4-HNE protein (*n* = 3 biologically independent experiments).

Western blot analysis confirmed that ICA69-/- mice with LPS significantly produced less COX2 and greater GPX4 in the cardiac tissues of these mice compared to those in the tissues of wild-type controls injected with LPS (Fig. 4A, B). In line with this, PTGS2 gene expression upregulated as expected, whereas intriguingly ICA69 did not affect the mRNA level of GPX4 (Fig. 4C, D). Lipid peroxidation could produce the fraction of lipid alkoxy, thus generating active aldehydes which include 4‐HNE and MDA [28]. LPS treatment resulted in elevated MDA in cardiac tissue and serum, which was decreased in LPS + ICA69 KO group (Fig. 4E, F). As expected, LPS gave rise to a significant decrease of the activity of superoxide dismutase (SOD), an antioxidase, which was almost reversed by ICA69 deficiency, but this change in serum was not statistically significant (Fig. 4G, H). Given that aberrant metabolism of iron acts as a vital ferroptosis factor, our research explored further the iron level in heart tissues and serum, both of which markedly rised twelve hours posterior to LPS treatment. ICA69 shortage evidently reversed the increased iron level in LPS mice (Fig. 4I, J), hinting that Fenton reactive process could be inhibited by ICA69 deficiency. While the upregulated levels of 4-HNE were caused by mutual effects between lipids and oxidant [29]. We next detected 4-HNE via immunohistochemical staining, and the results showed that LPS treatment gave rise to elevated 4-HNE in heart tissues, which were diminished by ICA69 deficiency (Fig. 4K). Collectively, this work demonstrates the crucial role of ICA69 in ferroptosis.

### ICA69 contributes to ferroptosis of LPS-induced RAW264.7 and H9C2 cells

**Fig. 5:**
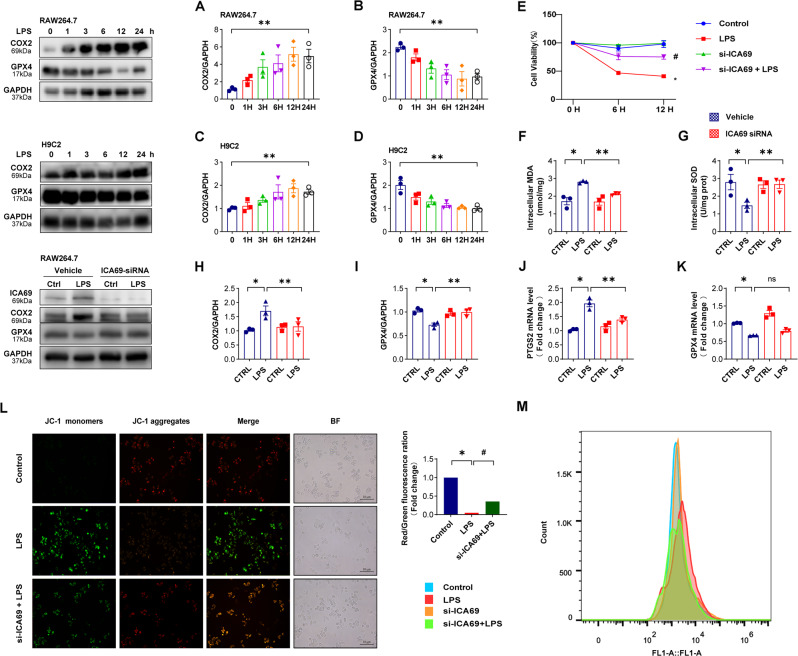
ICA69 contributes to ferroptosis of LPS-induced RAW264.7 and H9C2 cells. **A**, **B** Alterations of COX2 and GPX4 levels in RAW264.7 cells after LPS (1 μg/mL for 0 h, 1 h, 3 h, 6 h, 12 h, 24 h) treatment (*n* = 3). **C**, **D** Alterations of COX2 and GPX4 levels in H9C2 myofibroblasts after LPS (1 μg/mL for 0 h, 1 h, 3 h, 6 h, 12 h, 24 h) treatment. Levels of COX2 and GPX4 intensity were normalized to the corresponding GAPDH level, and the changes are shown in the histograms (*n* = 3). **P < 0.05 vs. 0 h group. **E** Cell viability was detected by an CCK8 assay in the indicated groups (*n* = 3). *P < 0.001 vs. Control group, #*P* < 0.05 vs. LPS group. **F** MDA levels in RAW264.7 cells stimulated by LPS (1 μg/mL) for 12 h after ICA69 siRNA transfection (*n* = 3). **G** The level of SOD in the indicated group RAW264.7 cells (*n* = 3). **H**, **I** The protein levels of COX2 and GPX4 in RAW264.7 cells transfected with ICA69 siRNA and then stimulated by LPS (1 μg/mL) for 12 h. Levels of COX2 and GPX4 intensity were normalized to the corresponding GAPDH level, and the changes are shown in the histograms (*n* = 3). **J**, **K** The mRNA levels of PTGS2 and GPX4 in RAW264.7 cells transfected with ICA69 siRNA and then stimulated by LPS (1 μg/mL) for 12 h (*n* = 3). The data are expressed as the mean ± standard error of the mean. *P < 0.05 vs. Vehicle + LPS group, **P < 0.05 vs. ICA69 siRNA + LPS group, ns, no significance. **L** JC-1 staining for mitochondrial membrane potential and cellular morphology under a phase-contrast microscope. (scale bar = 10 μm). **M** Intracellular ROS level was detected by flow cytometry.

Next, we found that after LPS stimulation for 0, 1, 3, 6, 12, and 24 h, the COX-2 protein level increased and GPX4 decreased over time in LPS-induced RAW264.7 cells (Fig. 5A, B), the protein expression of H9c2 myofibroblasts showed a similar alteration (Fig. 5C, D). To find out if ICA69 upregulation straightly participated in the ferroptosis induction, RAW264.7 cells were pretreated with ICA69 siRNA, before LPS. The cell viability assessment was performed to identify the impacts on ferroptosis in RAW264.7 cells challenged with LPS. Consistent with our prediction, siRNA-regulated ICA69 knockout offset LPS-caused growth suppression (Fig. 5E). Then western blot study verified that ICA69 siRNA markedly diminished COX-2 protein level, which was affected by LPS. In line with this, LPS markedly decreased GPX4 protein level, si-ICA69 reversed LPS-induced GPX4 inhibition as expected, whereas ICA69 did not affect the mRNA level of GPX4 (Fig. 5H–K). Previous work suggests that aberrant variation of mitochondrial membrane potential is more than a vital biomarker of mitochondrion injury, but an early sign of ferroptosis as well [30]. The outcomes (Fig. 5L) demonstrated that ICA69 suppression evidently decreased the fluorescence strength of JC-1 aggregates/JC-1 monomer probe in LPS-challenged RAW264.7 cells (1 μg/ml), signaling that ICA69 could elevate the mitochondrial membrane potential. The increased ROS is more than a vital reason for ferroptosis, but also the result posterior to it [31], our team discovered that ICA69 suppression could mitigate the rise of intracellular ROS caused by LPS (Fig. 5M). Moreover, our research also found that ICA69 deficiency offset as well the peroxidative effects in LPS macrophagocyte by decreasing the MDA level and increasing the SOD activity (Fig. 5F, G).

### xCT and GSH/GSGG levels in ICA69 deficiency mice during sepsis

**Fig. 6:**
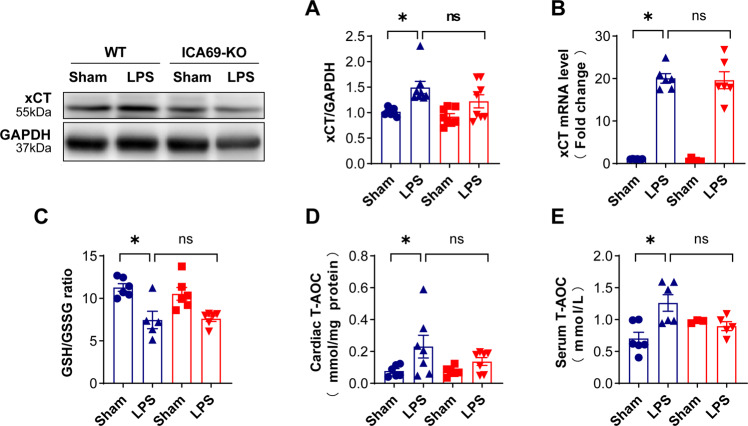
xCT and GSH/GSGG levels in ICA69 deficiency mice during sepsis. **A** Representative immunoblots and densitometric analysis of total xCT in cardiac tissue of ICA69-deficient (KO) and wild-type (WT) mice after LPS challenge. Levels of total xCT intensity were normalized to the corresponding GAPDH level, and the changes are shown in the histograms (*n* = 8). **B** The mRNA levels of xCT in the indicated groups that were stimulated with LPS or PBS for 12 h (*n* = 6). **C** GSH/GSSG ratio of ICA69-KO and WT mice after LPS injection (*n* = 6). **D**, **E** The level of T-AOC in the indicated groups in cardiac tissue and serum (*n* = 7/6). The data are expressed as the mean ± standard error of the mean. *P < 0.05 vs. WT + LPS group, ns no significance.

Besides, several ferroptotic events, including xCT (SLC7A11), are vital for modulating cystine absorption, maintenaning of intracellular cysteine and GSH pools and GSH depletion [32]. Western blots results exhibited that xCT, the substratum-specific subunit of an essential cystine/glutamate transporter, was notably increased in WT mice following LPS injection, and hardly varied compared with LPS + ICA69 KO group (Fig. 6A). Apart from the similar results in the gene expression level of xCT (Fig. 6B), we discovered that GSH, which is oxidized to GSH disulfide (GSSG) appears as a role in ROS neutralization [33, 34], with LPS being able to decrease the activities of glutathione peroxidase (GSH-Px) [35]. There are a variety of antioxidants in the body, including antioxidant macromolecules, antioxidant small molecules, and enzymes, which can remove various reactive oxygen species produced in the body to prevent the generation of oxidative stress induced by reactive oxygen species. The total antioxidant capacity (T-AOC) of a system is reflected by the total level of various macromolecules, small molecules, and enzymes in the system. We observed the consumed GSH/GSSG and T-AOC in four groups as mentioned earlier, and there is nOt evident variation in LPS + WT group and LPS + ICA69 KO group (Fig. 6C–E).

Collectively, this work demonstrates that xCT and GSH depletion are not essential for ICA69-associated ferroptosis.

### Increased ICA69 levels were positively correlated with the severity of sepsis

**Fig. 7:**
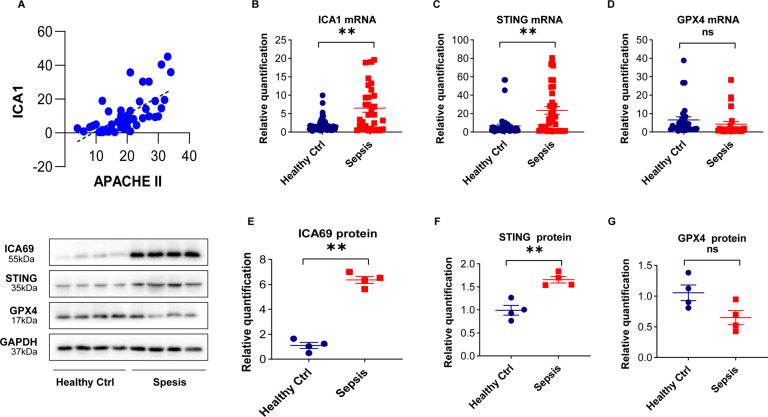
Increased ICA69 levels were positively correlated with the severity of sepsis. **A** Correlations of ICA69 expression with Acute Physiologic and Chronic Health Evaluation II scores in the septic patients (*r* = 0.6682, *P* < 0.001). Data were analyzed by Spearman correlation test. **B**–**D** The mRNA expression of ICA1, STING, and GPX4 were measured in PBMCs of 52 septic patients and 60 healthy controls by qRT-PCR. **E**–**G** ICA69, STING, and GPX4 levels were measured in PBMCs of septic patients and healthy controls by western blot. Relative expression was normalized to GAPDH levels. Corresponding histograms of the changes are shown (*n* = 4). The data are expressed as the mean ± standard error of the mean. **P < 0.05 vs. corresponding healthy controls, ns no significance.

ICA69, encoded by the ICA1 gene, in human is primarily dispersed in the pancreas, cerebrums, hearts, and thyroids. To reveal the significance of our discoveries, our team carried out an investigation of PBMCs from 52 cases hospitalized in ICUs, every one of which registered sepsis on admission, with an overall 38 survivals and 14 deaths in 28 days posterior to hospitalization. The baseline clinical features were determined via reviewing their electronic medical records and their ICA1 levels were documented in Table 1. The mean APACHE II value registered 19.5 ± 2.1, with 35 patients (67.3%) registering severe sepsis, 34 cases (65.4%) registering sepsis-induced shock, 34 cases (65.4%) undergoing ventilator therapy, and 7 cases (13.5%) undergoing renal replacement treatment. The most common infection regions were the abdomen and lung, and the region distribution resembled each other in survival and mortality group, the latter of which registered a notably higher ICA1 level (Table 1). The increased ICA1 level was affirmatively related to the sepsis severity, assessed via Acute Physiologic and Chronic Health Evaluation II scores (*r* = 0.6682, *P* < 0.001 Fig. 7A). Furthermore, the mRNA levels of ICA1 and STING were significantly increased, while GPX4 dropped in the septic group relative to the control (60 healthy controls and 52 septic patients) (Fig. 7B–D). The western blot results of ICA69, STING, and GPX4 were exhibited in Fig. 7E–G (4 sepsis cases and 4 normal controls). All four patients with sepsis shown here are male severe sepsis patients, who died within the first 28 days of intensive care unit (ICU) admission. We discovered that those data presented certain resemblance relative to the mouse experiments, highlighting that targeting ICA1 could serve as a cardioprotective strategy for cardiomyopathy prevention.

**Table 1:** Baseline clinical characteristics and ICA1 levels of the study subjects.

	Patient group			
Characteristics	All patients	Survivors	Nonsurvivors	*P* value
Demographics and underlying conditions				
Number of patients	52	38	14	
Males, number (%)	42 (80.8%)	32 (84.2%)	11 (78.6%)	–
Age (years)	67 ± 1.4	66 ± 34.6	69.9 ± 1.4	0.4639
Cardiac functional insufficiency, number (%)	20 (38.5%)	13 (34.2%)	7 (50%)	–
Hypertension, number (%)	17 (32.7%)	11 (28.9%)	6 (42.9%)	–
Type 2 Diabetes mellitus, number (%)	16 (30.8%)	10 (26.3%)	6 (42.9%)	–
Disease severity, number (%)				
Septic shock	34 (65.4%)	24 (63.2%)	10 (71.4%)	–
Baseline parameters				
APACHE II score	19.5 ± 2.1	16.8 ± 4.2	26.6 ± 2.1	0.000**
Site of infection, number (%)				
Lung	18 (34.6%)	12 (31.6%)	6 (42.9%)	–
Abdomen	21 (40.4%)	13 (34.2%)	8 (57.1%)	–
Urinary tract	8 (15.4%)	8 (21.1%)	0	–
Other	8 (15.4%)	5 (13.2%)	0	–
Intervention, number (%)				
Mechanical ventilation	34 (65.4%)	21 (55.3%)	13 (92.9%)	0.0614
Renal-replacement therapy	7 (13.5%)	5 (13.2%)	2 (14.3%)	0.7053
Length of stay				
In the ICU (days)	11.9 ± 24.7	10.8 ± 1.4	14.8 ± 24.7	0.0317*
In the hospital (days)	40.2 ± 56.6	47.3 ± 19.8	21 ± 6.6	0.0397*
ICA1	10.5 ± 3.5	7 ± 6.2	20.7 ± 3.5	0.000**

Data is presented as median (interquartile range); *p* value is analyzed by chi-square (gender, mortality, and site of infection), Kruskal–Wallis test (age and ICA1) or Mann–Whitney *U* test (APACHE II score).

*APACHE II* acute physiology and chronic health evaluation score.

*P < 0.05 survivors vs. nonsurvivors, **P < 0.001 survivors vs. nonsurvivors.

## Discussion

The prime discovery of our research is the participation of ICA69 in the pathogenesis of septic cardiac injury. The variations in ICA69 content regulated the STING expression, which further suppressed ROS-regulated inflammatory process and ferroptosis of cardiomyocytes.The knockout of ICA69 decreased cardiac damage and reversed survival ratios in murine experiments of LPS-induced sepsis. Furthermore, greater ICA69 level was identified in septic patients peripheralblood mononuclear cells (PBMCs) than in normal control groups. In this study, we identified that ICA69 can modulate the expression and activation of STING via directly combinating with it.

ICA69, which is heterogeneous, interacts with multiple antigens [3, 4] and possesses a unique framework encompassing BAR (Bin/Amphiphysin/Rvs) domains, realizing its mutual effect with different transporters to modulate protein trafficking and endocytosis [36, 37]. Previous research showed that, after contacting cGAMP or other cyclic dinucleotides, STING is stimulated and initiates ER-to-Golgi transfer by the translocon-related protein (TRAP) [13, 38–40], and then induces the expression of type I IFNs and proinflammatory cytokines (e.g., TNF and IL6) [41]. Thus, STING ER-to-Golgi trafficking is a vital initial phase in STING signaling and is a ratio-controlling step in pathway activation [10, 42]. Therefore, we have reason to speculate that ICA69, a positive regulator of STING signaling, recruited STING and promoted STING trafficking from the ER to the Golgi.

Previous research showed that LPS challenge could robustly elevate the expression of STING in macrophages [17]. Interestingly, another research presented that the level of STING protein expression was hardly influenced with diverse LPS dosage in neonatal rat cardiomyocytes (NRCMs) [12]. We suspect that, even though LPS did not affect the basal expression level of STING in Neonatal rat cardiomyocytes (NRCMs), it contributed to the activation of STING.

The latest research reveals that STING inhibition reduced the production of ROS and the lipid content of peroxidation products (4‐HNE and MDA) in murine lung challenged with LPS, and reversed the activity of antioxidase SOD [17]. Hence, we explored whether the upregulation of ICA69 participates in ferroptosis in LPS-induced septic cardiomyopathy. Our team unveiled that in the SIC progress caused by LPS, ICA69 deficiency reversed the levels of ferroptotic markers involving PTGS2, MDA, 4-HNE, GPX4, SOD, iron, and lipid ROS, but had no effect on the xCT-dependent manner. Attractively, we found that ICA69 can modulate the levels of iron in cardiac tissue and serum in LPS-treated mice. We speculate that ICA69 can participate in the endosomal/lysosomal degradation, which then interacts with ferritin and promotes its autophagic degradative process that hence produced massive free iron. Consequently, the current research unveils a fresh activation mechanism about ICA69 and provides a novel strategy for regulating ferroptosis.

GPX4 acts as a vital upstream ferroptosis mediator, which is a unique enzyme which safeguards cells from membrane lipid peroxidation and keeps redox homeostasis, via converting highly reactive lipid hydroperoxides (LOOH) into nonreactive lipid alcohols [24, 43]. One recent work shows that Gpx4 depletion activates the STING-dependent DNA sensor pathway [15]. In another work, the suppression of CTSB-related STING activation by RNAi blocks ferroptosis in cell cultivation and mouse models [16]. Jia M found that GPX4 shortage reinforces lipid peroxidation, which facilitates the carbonylation of STING and suppresses the ER-to-Golgi translocation in innate immune responses to DNA viruses but not to LPS [11]. On the basis of our experimental data (not shown), we found that ICA69 and STING can not directly combinate with GPX4. The exact interaction process between STING and GPX4 in LPS-induced mice needs to be further elucidated.

STING can lead to specific autophagy, in which a special cargo is identified and degraded [44–46]. Each autophagy-related protein doesn’t function identically all the time in immune activities, but exhibits notable regulatory role for the immune system [18]. As STING mutations related to human autoimmunity could induce constitutive ER exit and stimulate STING without cGAMP binding [42], the cGAS–STING pathway is pivotal for preventing massive infections and valid anti-tumor immune responses [47, 48]. Nevertheless, such pathway gives rise to the overmuch immune response which leads to the occurrence of certain autoinflammatory illnesses. Previous studies on ICA69 mainly focused on specific autoimmune diseases [3, 4]. Hence, more researches on the precise regulation of ICA69-STING complex might offer new perspective on STING-specific signaling and prevention of unwanted STING-independent process.

## Conclusions

In summary, we are the first to unveil that the knockout of ICA69 can weaken the LPS-caused cardiac damage via suppressing STING-mediated inflammation and ferroptosis. This research offers fundamental proof that ICA69 targeting might be of underlying treatment for septic cardiomyopathy.

## Materials and methods

### Reagents

Lipopolysaccharide (LPS) was bought from Sigma (St Louis, MO, USA). CCK-8 was bought from APExBIO Technology LLC (Los Angeles, USA). LDH assay kits were purchased from Nanjing Jiancheng Bioengineering Institute (Nanjing, China), and the assay kit of GSH/GSSG, MDA, SOD, T-AOC, JC-1, and ROS were purchased from Beyotime Biotechnology. Iron assay kit was acquired from Leagene Biotechnology Co., Ltd (Beijing, China), while ICA siRNA was acquired from Hanbio Biotechnology (Shanghai, China). Finally, other chemicals in our research met experimental requirements.

### Human sepsis samples

To observed human cellular activities from peripheral blood of normal subjects and septic cases, the entire consecutive sepsis patients hospitalized in the ICU between January 2021 and September 2021 were selected, with sepsis determined according to The Third International Consensus Definitions for Sepsis and Septic Shock [49, 50], whereas minor or pregnant patients, or those with type 1 diabetes or aplasia or immunosuppressive disease or those undergoing immunosuppressive treatments were not included in our research. On the ethics review, human study (ChiCTR-INR-2100051426) was accepted by the Ethics Committee of the First Affiliated Hospital of Wenzhou Medical University and was conformed to the protocols in the Declaration of Helsinki and in the Federal Policy for the Protection of Human Subjects, with informed consent provided by each participated patient. For further study, peripheral blood mononuclear cells (PBMCs) were acquired from blood by a density gradient centrifugation method (Ficoll Histopaque) [51].

### Animals

Male C57BL/6 mice and ICA69 KO mice (8–10 weeks old) were adopted, with the initial breeding pairs of ICA69−/− mice acquired from Ying Shen (Zhejiang University School of Medicine, Hangzhou) and kept at the Experimental Animal Center of Wenzhou Medical University. These animals were kept at 23 ± 1 °C with a twelve-hour light/dark period in a special disinfected environment with free access to bacteria-free water and food. Every animal assay, with a minimum of killed mice according to our design, was accepted by the Animal Experimentation Ethics Committee of Wenzhou Medical College(ID: wydw2019-0559). The entire experimental process was carried out in a blinded manner all along, involving the animal models and subsequent analyses.

The mice models with sepsis-caused cardiac muscle damage were established by intraperitoneal LPS injection (10 mg/kg) as per a past research [52], while the sham groups received injections intraperitoneally with isovolumetric PBS. After twelve hours, the mice received anaesthesia and transthoracic echocardiography to identify the heart functions. Subsequently, the mice were euthanized by exsanguination with overdose of sodium pentobarbital and every effort was made to minimize animals suffering. Blood samples in heparinized tubes from each group were centrifuged at 3000 × *g* for 15 min (4 °C). Meanwhile, the whole heart tissues were excised for histological analysis or snap-frozen in liquid nitrogen, stored at −80 °C for further biochemical detection.

### Cell culture and treatment

The murine monocyte/macrophage cell line RAW264.7 and H9C2 myofibroblasts were acquired from the Cell Bank of the Chinese Academy of Sciences (Shanghai, China). Cells were plated in dishes for 24 h, grown to 60–70% confluence. All siRNAs, purchased from Hanbio Biotechnology (Shanghai, China) and presented in Table 2, were used at a final concentration of 25 nM and ICA69 cDNA at 1 ng. The siRNAs transfected into cells with Lipofectamine™ RNAiMAX Transfection Reagent (Thermo Fisher Scientific) according to manufacturer’s instructions. With the cells transfecting over 24 h, LPS (1 μg/ml) was supplemented into the intermediate to establish a LPS-caused cardiomyocyte damage model in vitro. Posterior to LPS injection for 12 h, the cells placed in six-well plates were obtained to detect protein and analyze RNA in six-well plates by immunofluorescence dyeing observation. Meanwhile, patient-derived PBMCs obtained from the above-mentioned hospital were grown in RPM1640 (Gibco) with 10% FBS for further study. Generally, all cells were cultivated in an incubating device at 37 °C and 5% carbon dioxide. Specimens from one assay indicated an independent replicate, and there were at least three times of repetitions for all assays.

**Table 2:** Primers used for qRT-PCR/sequence of siRNAs.

Gene	Species	Forward (5′-3′)	Reverse (5′-3′)
ICA1	Human	CTTCGATCCCAAGGTTTCCAA	TCGACACAAAGGATTTCGTAAGG
STING	Human	CCAGAGCACACTCTCCGGTA	CGCATTTGGGAGGGAGTAGTA
GPX4	Human	GAGGCAAGACCGAAGTAAACTAC	CCGAACTGGTTACACGGGAA
GAPDH	Human	GGAGCGAGATCCCTCCAAAAT	GGCTGTTGTCATACTTCTCATGG
ICA1	Mouse	ACCACTCTGCTCCACTTTT	TTCTTCCCTTC TTTCTCAACT
TNFα	Mouse	ACTGAACTTCGGGGTGATCGGT	TGGTTTGCTACGACGTGGGCTA
IL-1β	Mouse	AATGAAGGAACGGAGGAGCC	CTCCAGCCAAGCTTCCTTGT
IL-6	Mouse	TAGTCCTTCCTACCCCAATTTCC	TTGGTCCTTAGCCACTCCTTC
STING	Mouse	GGTCACCGCTCCAAATATGTAG	CAGTAGTCCAAGTTCGTGCGA
IFN-β	Mouse	TGTCTGCGAGCCTAGAGACTA	AGCCGGGAATTTCGTATTGTTAT
PTGS2	Mouse	TTCAACACACTCTATCACTGGC	AGAAGCGTTTGCGGTACTCAT
GPX4	Mouse	CCTCTGCTGCAAGAGCCTCCC	CTTATCCAGGCAGACCATGTGC
xCT	Mouse	GGCACCGTCATCGGATCAG	CTCCACAGGCAGACCAGAAAA
GAPDH	Mouse	ACTCCACTCACGGCAAATTC	TCTCCATGGTGGTGAAGACA
mmu-ica69-si		CCGAGGCAGUUAAUUUCUUTT	AAGAAAUUAACUGCCUCGGTT
mmu-GAPDH-si		GUGGAGAUUGUUGCCAUCATT	UGAUGGCAACAAUCUCCACTT

*qRT-PCR* quantitative real-time polymerase chain reaction.

### Cell viability assay

With cytoactive identified by CCK-8 method (APExBIO Technology LLC, Los Angeles, USA), RAW264.7 and H9C2 cells were cultivated in 96-well plates at 10^5 cells/mL and exposed to different concentrations of LPS (1 μg/mL) or 0.1% DMSO as a control for 0, 6, 12 h and 10 μL of CKK8 was supplemented into each well for two-hour incubation. Eventually, the absorbance at 450 nm was detected on a microplate reader, while abundance was measured by three wells of every specimen, and all assays were carried out in triplicate.

### Western blotting

The total proteins in whole frozen cardiac tissues and iced cell lysates were extracted and quantified, 50 μg proteins were treated with 100 °C for five minutes. Equal amounts of proteins were separated by 10–15% SDS-PAGE. Samples were transferred to polyvinylidene difluoride membranes, blocked with 5% dry skim milk, and incubated with primary antibodies including anti-ICA69 (SANTA CRUZ, sc-271489, 1:1000), anti-ICA69 (Abcam, Ab154391, 1:1000), anti-GPX4 (Abcam, Ab125066, 1:1000), anti-GAPDH (Abcam, Ab181602, 1:2000), anti-Sting (CST, D2P2F, 1:1000), anti-Tubulin beta Antibody (Affinity, AF7011, 1:5000), anti-COX2/PTGS2 (Abcam, Ab179800, 1:1000), and anti-xCT(Abcam, Ab175186, 1:1000), separately, at 4 °C nightlong. Proteins in western blot were quantified via Image Lab software.

### Coimmunoprecipitation

Homogenized cardiac tissues were acquired and lysed. After the centrifugal process, the supernatant was adopted as the input, whereas the residual was leveraged for coimmunoprecipitation(CO-IP). Our team added 10 µL Mouse anti-ICA69 antibody (SANTA CRUZ, sc-271489) and 20 µL resuspended Protein A/G PLUS-Agarose cultivated under 4 °C on a rocker device or rotating equipment overnight to harvest immunoprecipitates using the centrifuge. We wash the pellet by 1.0 mL RIPA buffer for four times, repeating the centrifugal process every time. When the final wash was completed, we pumped and deserted the supernatant and resuspended the pellet in 40 µL of 2×nuPAGE loading buffer and boiled for 5 min at 100 °C.

### Histological analysis

Hematoxylin and eosin staining(Solarbio, Beijing, China) were implemented as per our past research. Specimens of fresh cardiac tissues were fixed in 4% paraformaldehyde and sliced into films of 5 μm. The heart samples sliced from the central segment were dyed by H&E for the evaluation of myofilament morphological status and inflammation cell infiltration.

For immunohistochemistry (IHC), paraffin sections were deparaffinized with xylene and rehydrated with the concentration gradient of ethanol. Then, antigen retrieval was conducted and the samples were incubated with primary antibodies for 4HNE (Abcam, ab48506, 1:200) nightlong at 4 °C. Eventually, the slices were cultivated by an anti-rabbit EnVisionTM + /HRP reagent for fifty minutes at 37 °C for the observation via light microscopy [Nikon (Tokyo, Japan), H550L].

### Immunofluorescence staining

The cardiac ventricle tissues were harvested at 12 h posterior to LPS and fixed in 4% paraformaldehyde for four to six hours at 4 °C, transferred to 15% sucrose liquor at 4 °C at the bottom, subsequently transferred to 30% sucrose liquor at 4 °C till sinking. Furthermore, tissue was embedded with optimum cutting temperature compound (OCT) (Solarbio, Shanghai, China) at −20 °C and maded into 8 μm slices with a cryostat, while samples or cell coverslips were treated with formaldehyde at a 4% concentration for fixation and then permeabilized in 0.2% TritonX-100 and 10% goat serum for a whole hour under 37 °C. Subsequently, samples or coverslips were cultivated by the primary antibodies anti-ICA69 (Santa, sc-271489, 1:100) or anti-CD68 (CST, D4B9C, 1:100) at 4 °C nightlong. The next day, the Alexa Fluor 488-goat anti-mouse and 594-goat anti-rabbit secondary antibodies (1:200) were used to probe target proteins for a whole hour free of light. Eventually, the nuclei were cultured by DAPI (Invitrogen, CA, USA) at ambient temperature for five minutes, and the specimens were studied via a Leica Sp5 II laser confocal microscope for the observation of the expression and location of protein.

### Quantitative real-time PCR

Overall RNA from the whole frozen cardiac tissues and iced cell were extracted by Trizol (Invitrogen), and reversely transcribed into cDNA by a cDNA synthesis kit (Takara Clontech, Dalian, Japan). As quantitative PCR was performed by a Step one plus Real-Time PCR system (Applied Biosystems, USA) via gene-specific primers, the RNA quantity was computed via the comparative threshold cycle approach, with every primer customized by Sangon Biotechnology Co., Ltd. (Shanghai, China). Eventually, the each primer sequence is presented in Supplementary Table 2.

### Flow cytometry assay for ROS

RAW264.7 cells were implanted at 2 × 105/well in 12-well plates, activated by LPS (1 μg/mL) for 12 hours to detect the intracellular total ROS using a Reactive Oxygen Species Assay kit as per the supplier’s specification. After treatment, the cell cultivation intermediate was cleared, and dichloro-dihydro-fluorescein diacetate (DCFH-DA) was supplemented to an eventual 10 µM. Afterwards, the cells were cultured in a carbon dioxide incubation device at 37 °C for 20 min and cleaned by PBS three times to fully clear the DCFH-DA from them. With these completed, RAW264.7 cells were obtained via centrifugal technique, aspirating and discarding the supernatant, and resuspending with PBS, while DCF fluorescence intensities were detected by flow cytometry or a multidetection reader at excitation and emission wavelengths of 485 nm and 535 nm, separately. Consequently, the level of ROS in all specimens was analyzed by FlowJo Software. Finally, each sample was assayed in triplicate to ensure the authenticity of the experiments.

### JC-1 staining

Cell concentration and grouping resembled their counterparts in the above mentioned intracellular ROS assay. As mitochondrial membrane potential in RAW264.7 cells was determined via JC-1 fluorescent probe, these cells were cultivated by JC-1 working solution for 20 minutes at 37 °C after treated with LPS(1 μg/mL) for 12 h. Afterwards, JC-1 buffer liquor was applied to clean the cells three times at least. The outcomes were depicted by the rate of the green/red fluorescence strength, who referred to the level of mitochondrion disruption.

### Assessment of oxidative stress

our team carried out the specimen preparation as per the assay kit specification. The levels of malondialdehyde (MDA) [53], superoxide dismutase (SOD) [54], GSH/GSSG [55], total antioxidant capacity (T-AOC) [56] in serum and heart samples were measured via colorimetric determination by assay kits according to previous studies mentioned above. The results of MDA, SOD were expressed as a unit per mg protein (U/mg prot).

### Iron in serum and cardiac tissue

The tissue weight was accurately weighed, while the homogenate was mechanically prepared in an ice bath at 2500 rpm for 10 min to produce a 10% supernatant. After the sample is prepared, the protein level can be detected by the BCA Protein Concentration Assay Kit to facilitate the subsequent calculation of Fe content in tissues or cells per unit protein weight. Afterwards, iron standard test sample and iron test base liquid were added in sequence. Eventually, we mix the well, while detecting the standard well at 562 nm with the enzyme plate analyzer, measuring the well absorbance, and finish the colorimetry within 1 h.

### LDH and CK MB in cardiac tissue

The quantity of cardiac tissue creatine kinase isoenzyme (CK-MB) was determined by a biochemical analyzing machine automatically (ADVIA® 2400, Siemens Ltd., China). The enzyme activities of lactate dehydrogenase (LDH) in serum were identified by rapid and sensitive assay kits according to the instruction. Briefly, we produced the specimens for the standard curve by nicotinamide adenine dinucleotide mother liquor and LDH buffer. A 50 μL Reaction Mix with 48 μL LDH Assay Buffer in it and 2 μL LDH Substrate Mix was supplemented into the specimens or standard specimens for a whole hour at 37 °C free of light, producing a 450 nm absorbance.

### Survival condition

The extra mice in all groups (*n* = 10) were raised to study the survival status. The mortality was daily documented at the identical time node, while the survival rate was computed within seven days posterior to LPS injection at 10 mg/kg or PBS.

### Echocardiography

Echocardiography was implemented by a Vevo 3100 ultrasonic equipment with a 10-MHz linear array ultrasound transducer (Fujifilm, VisualSonics, USA) after mice were anesthetized by 1.5% isoflurane. As the medial echocardiographic readings were collected from 3–5 heart cycles, the heart function indexes, such as fractional shortening (FS), heart rate (bpm), ejection fraction (EF), etc., were documented.

### Statistical analysis

The entire measured data here were depicted by average ± SEM or characteristic images of 1 representative from 3 separate assays. As GraphPad Prism 8.0.2 software for Windows was adopted for statistic observation, the comparison between the two groups was performed by Student’s t-test, and the diversities between the groups were compared by two-way ANOVA and corrected by Bonferroni, with survival condition assessed by Kaplan–Meier analysis. Human data were studied by Wilcoxon (Exact) rank-sum test, while association among the expression of ICA1 in PBMCs and Acute Physiology and Chronic Health Evaluation II value of septic cases was evaluated by the Pearson correlation analysis. A *P* < 0.05 was deemed as significant on statistics.

## Supplementary information

Original Western Blots

Ethical Inspection ID ChiCTR-INR-2100051426

Informed Consent Form

Clinical Research and Ethical Review

Ethical Inspection ID wydw2019-0559

## Data Availability

The datasets adopted in our research are accessible from the relevant author on reasonable request.
