# Supplementary material for: ICA69 aggravates ferroptosis causing septic cardiac dysfunction via STING trafficking
Source: Cell Death Discov. 2022 Apr 9;8:187. doi: 10.1038/s41420-022-00957-y (PMC8994779; doi:10.1038/s41420-022-00957-y)

Western blots Original source data

**Figure 1A: ICA69-GAPDH (Mouse)**

*** [Anti-ICA69 antibody (SANTA CRUZ, sc-271489, 1:1000)](https://www.abcam.cn/ica69-antibody-ab154391.html);**

**[Anti-GAPDH antibody (Abcam, Ab181602, 1:2000)](https://www.abcam.cn/gapdh-antibody-epr16891-loading-control-ab181602.html)**

**
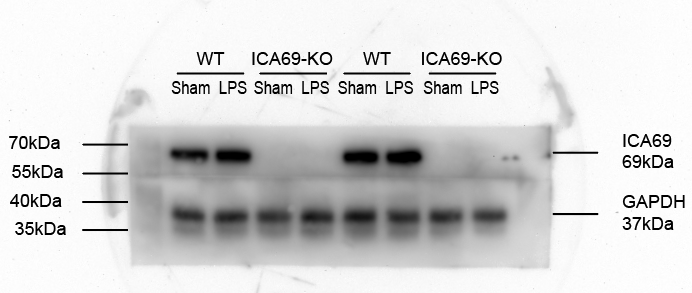
N=1; N=2**

**N=3; N=4**

**
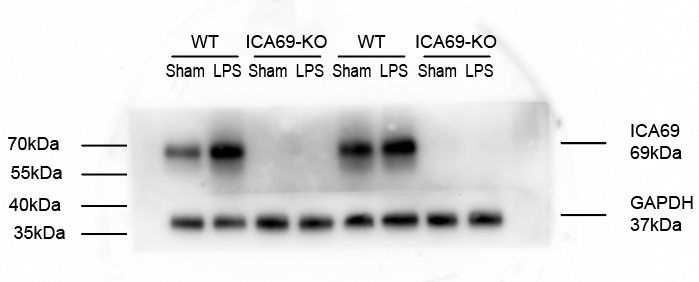
**

**Figure 1A: ICA69-GAPDH (Mouse)**

*** [Anti-ICA69 antibody (SANTA CRUZ, sc-271489, 1:1000)](https://www.abcam.cn/ica69-antibody-ab154391.html);**

**[Anti-GAPDH antibody (Abcam, Ab181602, 1:2000)](https://www.abcam.cn/gapdh-antibody-epr16891-loading-control-ab181602.html)**

**N=5**


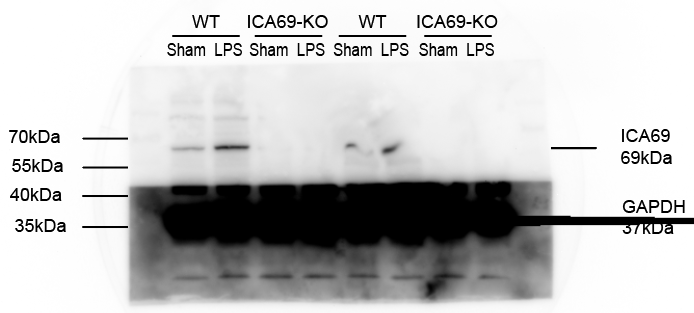

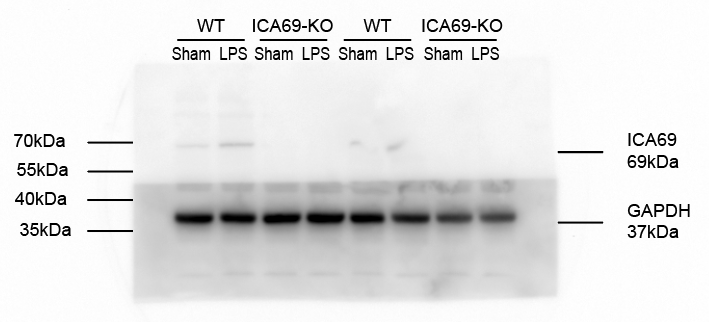


**Figure 1A: ICA69-GAPDH (Mouse)**

*** [Anti-ICA69 antibody (SANTA CRUZ, sc-271489, 1:1000)](https://www.abcam.cn/ica69-antibody-ab154391.html);**

**[Anti-GAPDH antibody (Abcam, Ab181602, 1:2000)](https://www.abcam.cn/gapdh-antibody-epr16891-loading-control-ab181602.html)**

**N=6**


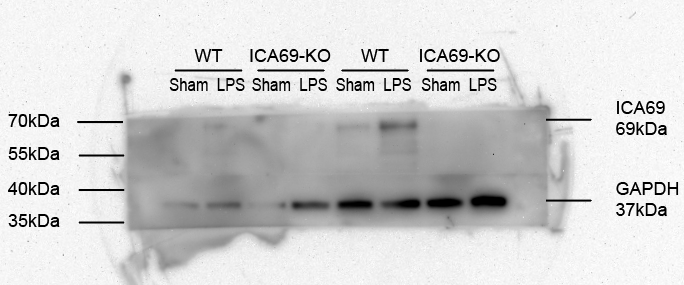


**N=7; N=8**

**
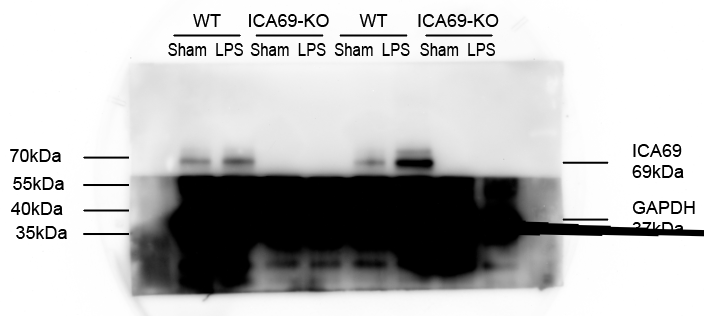

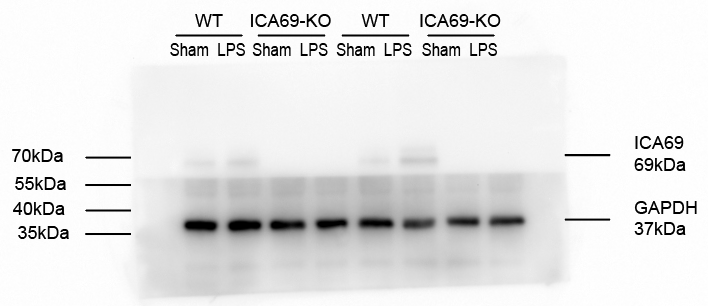
**

**Figure 1C: ICA69-GAPDH (RAW264.7 Cell)**

*** [Anti-ICA69 antibody (SANTA CRUZ, sc-271489, 1:1000)](https://www.abcam.cn/ica69-antibody-ab154391.html);**

**[Anti-GAPDH antibody (Abcam, Ab181602, 1:2000)](https://www.abcam.cn/gapdh-antibody-epr16891-loading-control-ab181602.html)**

**N=1; N=2**


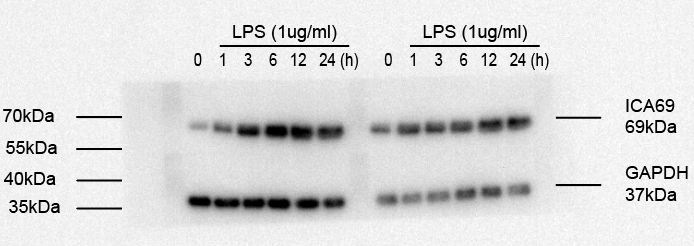


**N=3**


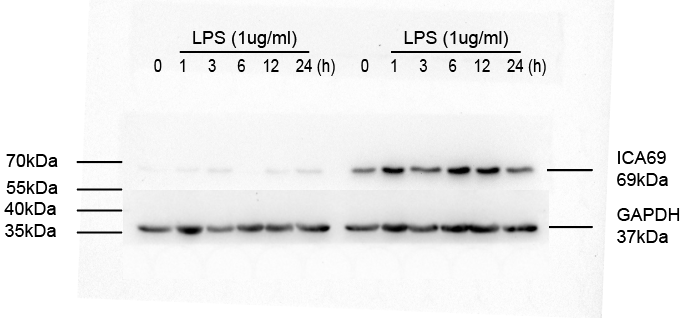


**N=4**

**
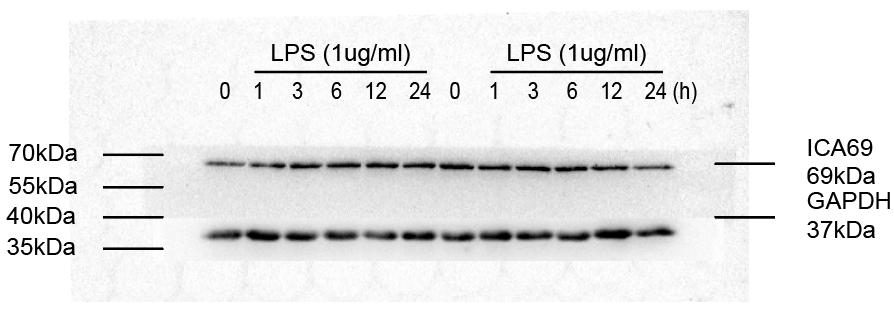
**

**Figure 1D: ICA69-GAPDH (H9c2 Cell)**

*** [Anti-ICA69 antibody (SANTA CRUZ, sc-271489, 1:1000)](https://www.abcam.cn/ica69-antibody-ab154391.html);**

**[Anti-GAPDH antibody (Abcam, Ab181602, 1:2000)](https://www.abcam.cn/gapdh-antibody-epr16891-loading-control-ab181602.html)**

**N=1; N=2**

**
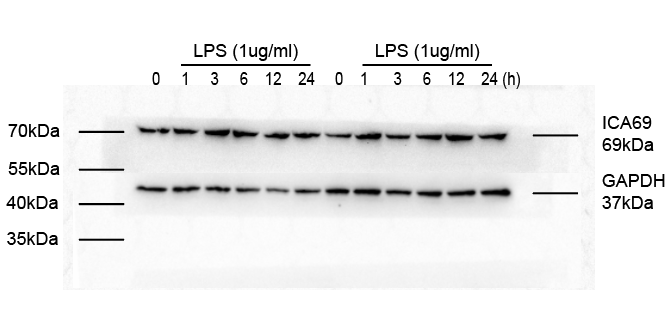
**

**N=3; N=4**

**
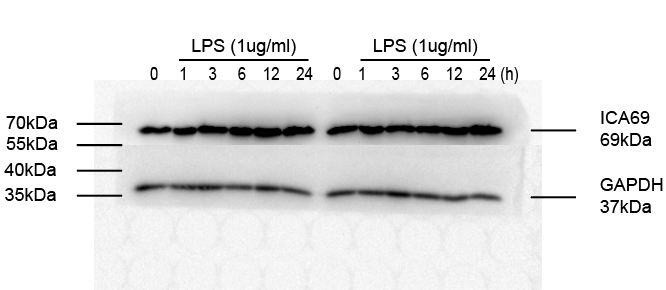
**

**Figure 3A: TUBULIN-STING (Mouse)**

***Anti-[Tubulin beta Antibody](http://www.affbiotech.cn/goods-6286-AF7011-Tubulin+beta+Antibody.html) (Affinity, [AF7011](http://www.affbiotech.cn/goods-6286-AF7011-Tubulin+beta+Antibody.html), 1:5000)**

**Anti-STING antibody (CST, D2P2F, 1:1000)**

**N=1; N=2**


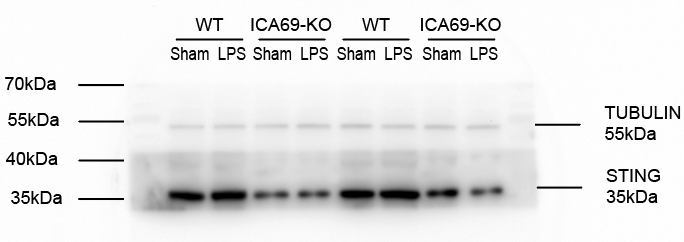


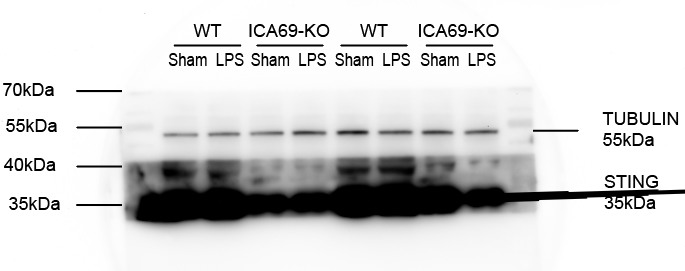


**Figure 3A: TUBULIN-STING (Mouse)**

***Anti-[Tubulin beta Antibody](http://www.affbiotech.cn/goods-6286-AF7011-Tubulin+beta+Antibody.html) (Affinity, [AF7011](http://www.affbiotech.cn/goods-6286-AF7011-Tubulin+beta+Antibody.html), 1:5000)**

**Anti-STING antibody (CST, D2P2F, 1:1000)**

**N=3; N=4**


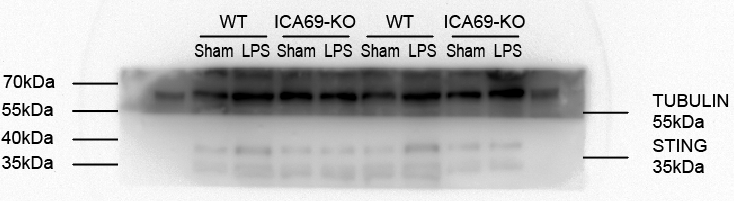


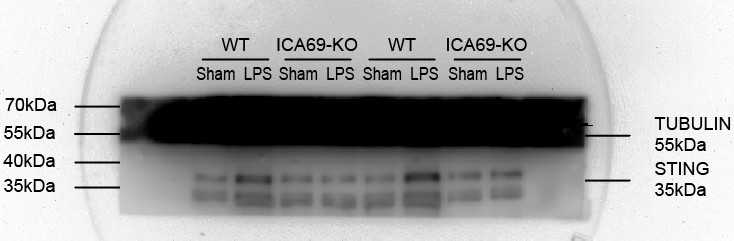


**
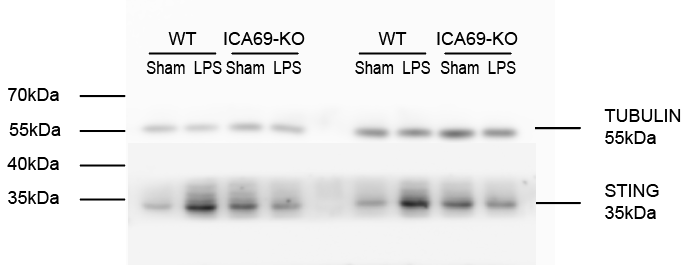
**

**N=5; N=6**

**Figure 4A-B: ICA69-KO: COX2-GAPDH-GPX4 (Mouse)**

***[Anti-COX2 / PTGS2 antibody (Abcam, Ab179800, 1:1000)](https://www.abcam.cn/cox2--cyclooxygenase-2-antibody-epr12012-ab179800.html);**

**[Anti-GAPDH antibody (Abcam, Ab181602, 1:2000)](https://www.abcam.cn/gapdh-antibody-epr16891-loading-control-ab181602.html);**

**Anti-GPX4 (Abcam, Ab125066, 1:1000)**

**N=1; N=2**


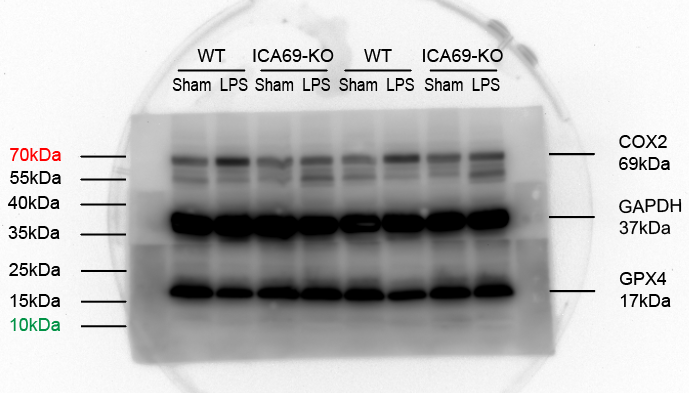


**N=3; N=4**

**
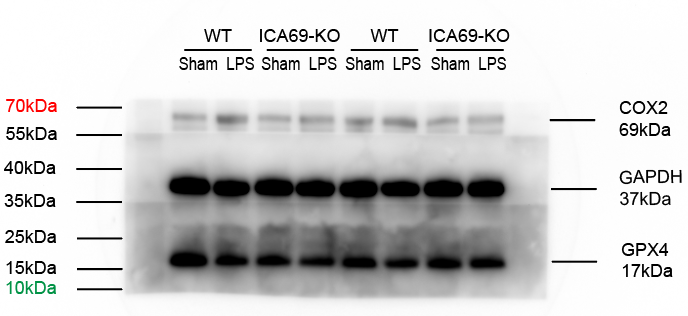
**

**Figure 4A-B: ICA69-KO: COX2-GAPDH-GPX4 (Mouse)**

***[Anti-COX2 / PTGS2 antibody (Abcam, Ab179800, 1:1000)](https://www.abcam.cn/cox2--cyclooxygenase-2-antibody-epr12012-ab179800.html);**

**[Anti-GAPDH antibody (Abcam, Ab181602, 1:2000)](https://www.abcam.cn/gapdh-antibody-epr16891-loading-control-ab181602.html);**

**Anti-GPX4 (Abcam, Ab125066, 1:1000)**

**N=5; N=6**


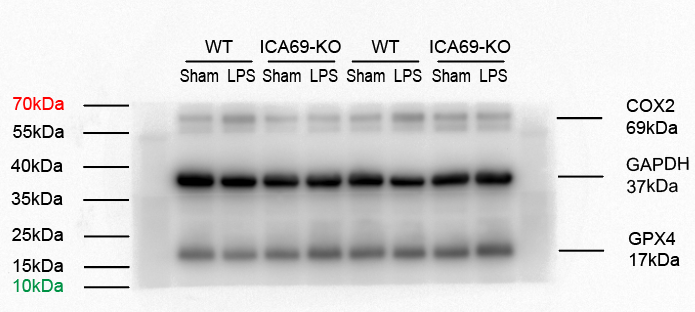


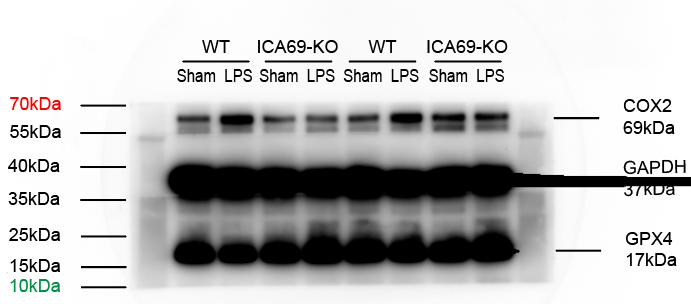


**Figure 5A-B: COX2-GAPDH-GPX4 (RAW264.7 Cell)**

***[Anti-COX2 / PTGS2 antibody (Abcam, Ab179800, 1:1000)](https://www.abcam.cn/cox2--cyclooxygenase-2-antibody-epr12012-ab179800.html);**

**[Anti-GAPDH antibody (Abcam, Ab181602, 1:2000)](https://www.abcam.cn/gapdh-antibody-epr16891-loading-control-ab181602.html);**

**Anti-GPX4 (Abcam, Ab125066, 1:1000)**

**N=1**


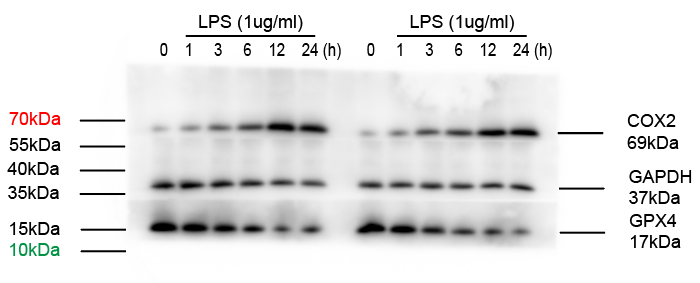


**N=2**


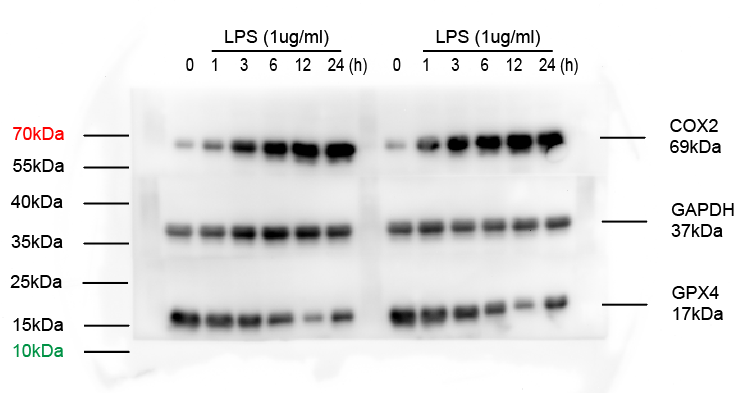


**Figure 5A-B: COX2-GAPDH-GPX4 (RAW264.7 Cell)**

***[Anti-COX2 / PTGS2 antibody (Abcam, Ab179800, 1:1000)](https://www.abcam.cn/cox2--cyclooxygenase-2-antibody-epr12012-ab179800.html);**

**[Anti-GAPDH antibody (Abcam, Ab181602, 1:2000)](https://www.abcam.cn/gapdh-antibody-epr16891-loading-control-ab181602.html);**

**Anti-GPX4 (Abcam, Ab125066, 1:1000)**

**N=3**

**
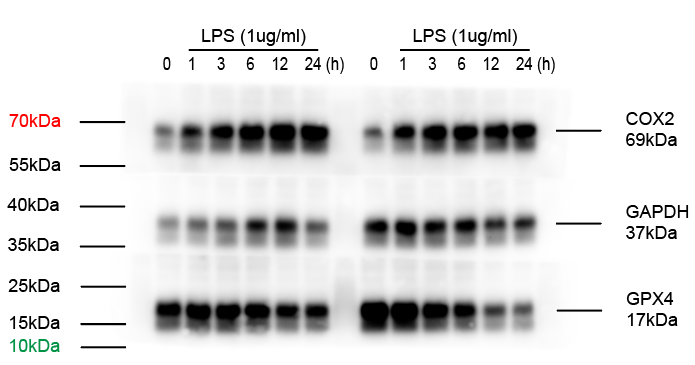
**

**Figure 5C-D: COX2-GAPDH-GPX4 (H9c2 Cell)**

***[Anti-COX2 / PTGS2 antibody (Abcam, Ab179800, 1:1000)](https://www.abcam.cn/cox2--cyclooxygenase-2-antibody-epr12012-ab179800.html);**

**[Anti-GAPDH antibody (Abcam, Ab181602, 1:2000)](https://www.abcam.cn/gapdh-antibody-epr16891-loading-control-ab181602.html);**

**Anti-GPX4 (Abcam, Ab125066, 1:1000)**


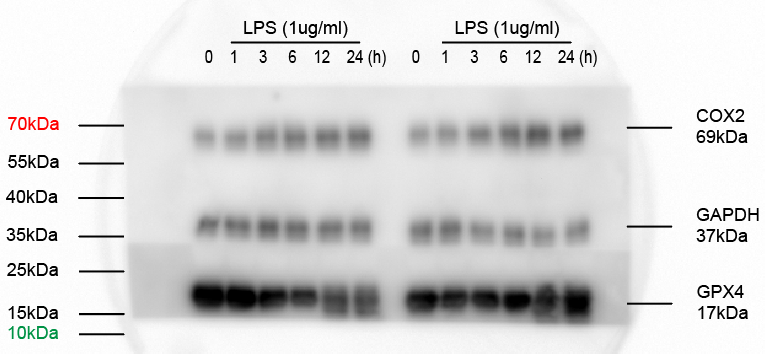
**N=1**

**N=2**

**
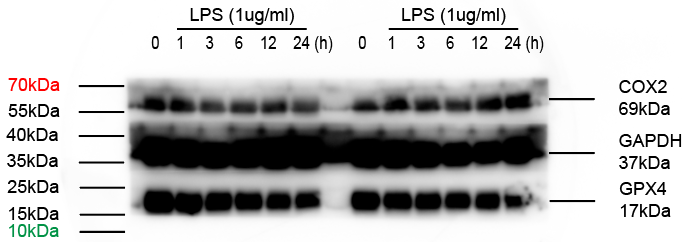
**
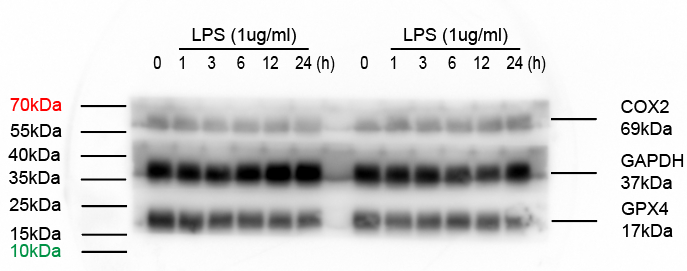


**Figure 5C-D: COX2-GAPDH-GPX4 (H9c2 Cell)**

***[Anti-COX2 / PTGS2 antibody (Abcam, Ab179800, 1:1000)](https://www.abcam.cn/cox2--cyclooxygenase-2-antibody-epr12012-ab179800.html);**

**[Anti-GAPDH antibody (Abcam, Ab181602, 1:2000)](https://www.abcam.cn/gapdh-antibody-epr16891-loading-control-ab181602.html);**

**Anti-GPX4 (Abcam, Ab125066, 1:1000)**

**N=3**

**
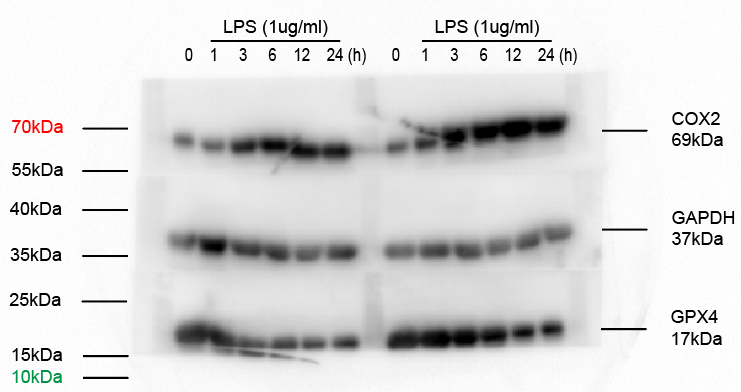
**

**Figure 5H-I: si-ICA69: COX2-GAPDH-GPX4 (RAW264.7 Cell)**

***[Anti-COX2 / PTGS2 antibody (Abcam, Ab179800, 1:1000)](https://www.abcam.cn/cox2--cyclooxygenase-2-antibody-epr12012-ab179800.html);**

**[Anti-GAPDH antibody (Abcam, Ab181602, 1:2000)](https://www.abcam.cn/gapdh-antibody-epr16891-loading-control-ab181602.html);**

**Anti-GPX4 (Abcam, Ab125066, 1:1000)**

**N=1**


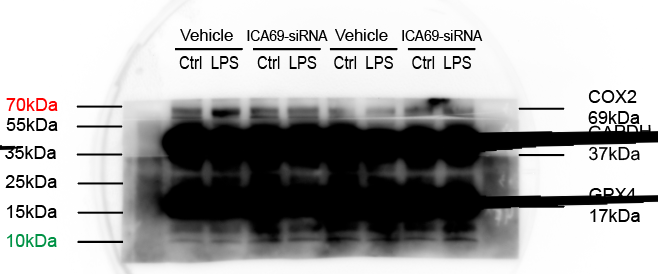

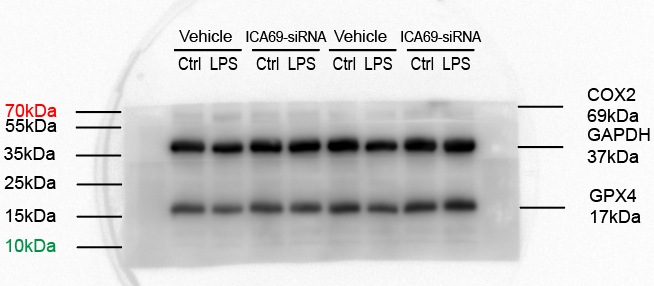


**Figure 5H-I: si-ICA69: COX2-GAPDH-GPX4 (RAW264.7 Cell)**

***[Anti-COX2 / PTGS2 antibody (Abcam, Ab179800, 1:1000)](https://www.abcam.cn/cox2--cyclooxygenase-2-antibody-epr12012-ab179800.html);**

**[Anti-GAPDH antibody (Abcam, Ab181602, 1:2000)](https://www.abcam.cn/gapdh-antibody-epr16891-loading-control-ab181602.html);**

**Anti-GPX4 (Abcam, Ab125066, 1:1000)**

**N=2**


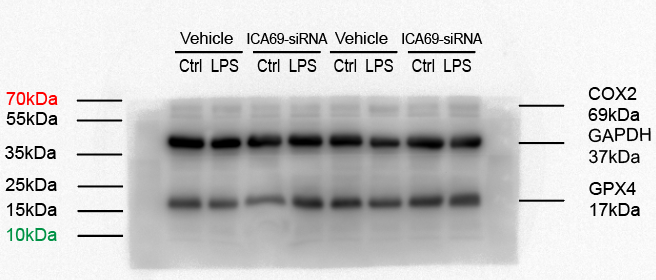


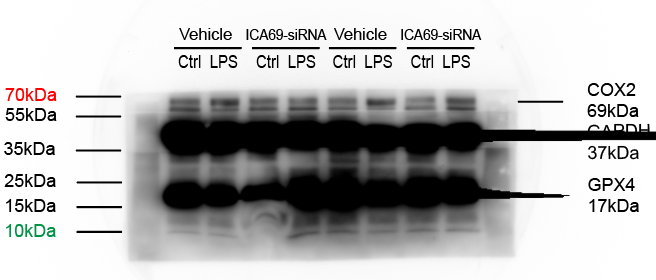


**Figure 5H-I: si-ICA69: COX2-GAPDH-GPX4 (RAW264.7 Cell)**

***[Anti-COX2 / PTGS2 antibody (Abcam, Ab179800, 1:1000)](https://www.abcam.cn/cox2--cyclooxygenase-2-antibody-epr12012-ab179800.html);**

**[Anti-GAPDH antibody (Abcam, Ab181602, 1:2000)](https://www.abcam.cn/gapdh-antibody-epr16891-loading-control-ab181602.html);**

**Anti-GPX4 (Abcam, Ab125066, 1:1000)**

**N=3**


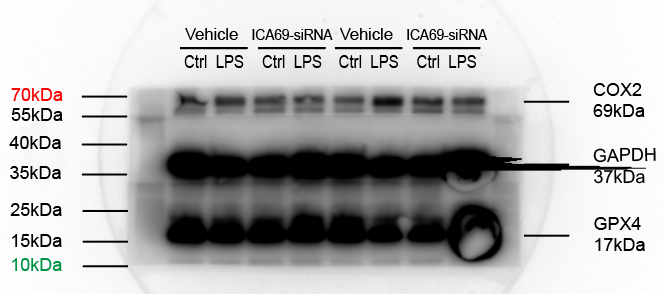

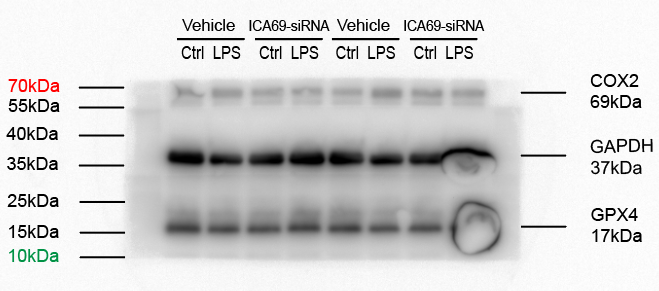


**Figure 6A-B: ICA69-KO: xCT-GAPDH (Mouse)**

***[Anti-xCT antibody (Abcam, Ab175186, 1:1000)](https://www.abcam.cn/xct-antibody-epr82902-ab175186.html)**

**[Anti-GAPDH antibody (Abcam, Ab181602, 1:2000)](https://www.abcam.cn/gapdh-antibody-epr16891-loading-control-ab181602.html)**

**
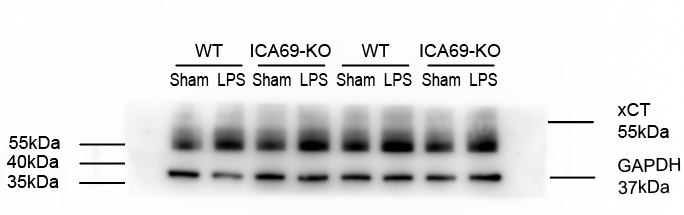
N=1; N=2**

**N=3; N=4**

**
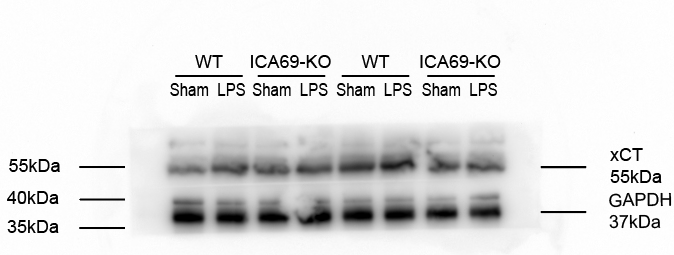
**

**N=5; N=6**

**
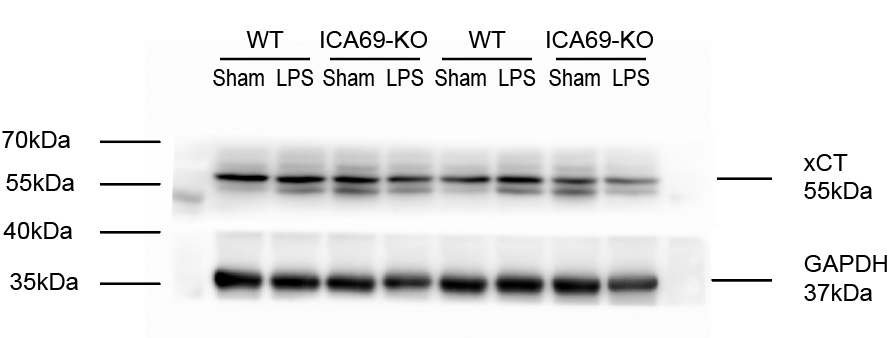
**

**Figure 7E &G: ICA69-GAPDH-GPX4 (Human)**

***[Anti-ICA69 antibody (Abcam, Ab154391, 1:1000)](https://www.abcam.cn/ica69-antibody-ab154391.html);**

**[Anti-GAPDH antibody (Abcam, Ab181602, 1:2000)](https://www.abcam.cn/gapdh-antibody-epr16891-loading-control-ab181602.html);**

**Anti-GPX4 (Abcam, Ab125066, 1:1000)**

**N=1-4**

**
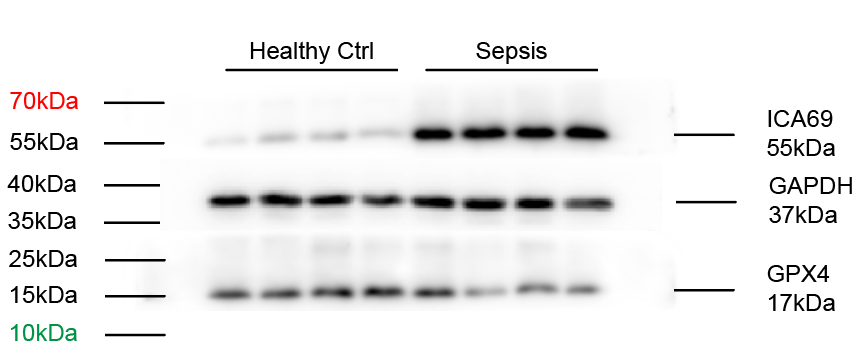
**

**Figure 7F: STING (Human)**

**Anti-STING antibody (CST, D2P2F, 1:1000)**

**N=1-4**


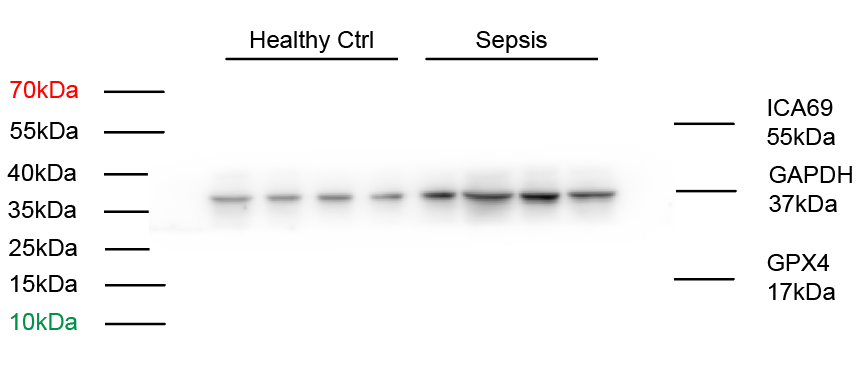

Supplement: Supplementary file 1 — Original Western Blots [file 41420_2022_957_MOESM1_ESM.docx]
