# Supplementary material for: ICA69 aggravates ferroptosis causing septic cardiac dysfunction via STING trafficking
Source: Cell Death Discov. 2022 Apr 9;8:187. doi: 10.1038/s41420-022-00957-y (PMC8994779; doi:10.1038/s41420-022-00957-y)
Supplement: Supplementary file 2 — Ethical Inspection ID ChiCTR-INR-2100051426 [file 41420_2022_957_MOESM2_ESM.pdf]

温州医科大学附属第一医院临床研究伦理委员会审查批件

（Review of Ethics Committee in Clinical Research (ECCR) of the First Affiliated Hospital of Wenzhou Medical University）

受理编号 Acceptance Number：KY2021-105　　批件号：临床研究伦审 Issuing Number（2021）第（105）号

项目名称 Project	ICA69 调节脓毒症性心功能障碍的分子机制研究 Molecular mechanism of ICA69 in regulating sepsis-induced cardiomyopathy in mice		
申办者 Applicant	温州医科大学附属第一医院	试验目的 Objective	临床科研 Clinical research
试验科室 Department	麻醉科		
试验项目负责人 Principal Investigator	王均炉		
审查方式和时间 Form and Date	☐ 会议审查 Review Conference，时间：＿＿＿＿<br>☑ 快速审查 Fast track，时间：2021 年 6 月 24 日		
审查地点 Review Site	新院 1-4A18 会议室		
审查材料 Documents for Review	1、医学临床科研项目及伦理审查申请表，v1.0 版；<br>2、临床研究方案，v1.0 版，2021.01.30；<br>3、受试者知情同意书，v1.0 版,2021.01.30；<br>4、研究者团队成员目录（职责）；<br>5、主要研究者、团队成员简历及 GCP 证书，v1.0 版；<br>6、研究者责任声明；<br>7、CRF/临床观察表样板，v1.0 版。		
审查意见 Comments	根据国家卫健委《涉及人的生物医学研究伦理审查办法》(2016)、WMA《赫尔辛基宣言》和 CIOMS《人体生物医学研究国际道德指南》的伦理原则，经本伦理委员会审查，**同意**该项目开展。<br>According to the Regulations and Rules of "Ethical Reviews for Biomedical Research Involving Human Subjects" (2016) the National Health Commission of PRC , "Declaration of Helsinki" of WMA, and "International Ethical Guidelines for Human Biomedical Research" of CIOMS , the project was **approved** by **ECCR** .		
主任委员/副主任委员签字 Signature of the **ECCR** Chair	[signature]	签发日期 Date	2021.6.25
温州医科大学附属第一医院临床研究伦理委员会（盖章）Ethics Committee in Clinical Research of the First Affiliated Hospital of Wenzhou Medical University (Seal)			

温州医科大学附属第一医院临床研究伦理委员会专用章

版本日期：2021 年 06 月 21 日

附注（Note）：

1. 临床研究应在批准之日起 1 年内实施，逾期未实施，本批件自行废止。临床研究过程中将接受伦理委员会的跟踪审查，审查频度为自批准之日起每 12 个月一次。（伦理委员会有权根据临床试验实际开展情况改变跟踪审查频度）
   The clinical study shall be implemented within 1 year from the date of approval. If overdue, the approval for this project shall be revoked. During the implementation of clinical research, tracking review will be conducted by **ECCR** every 12 months from the effective date of the initial approval (the ethics committee has the right to change the frequency of tracking review according to the actual implementation of clinical trials).
2. 请严格遵从已批准的研究方案，如果方案修改需以书面形式报告伦理委员会，经伦理委员会批准后方可执行。
   Please strictly follow the approved research protocol. Any revisions of the protocol must be reported to **ECCR** in written form. It can be conducted only after the modification was approved by **ECCR**.
3. 发生严重不良事件以及影响研究风险受益比的非预期不良事件，须在 24 小时内报告本伦理委员会。
   Serious adverse events and unanticipated adverse events that affect the risk-to-benefit ratio of the project must be reported to **ECCR** within 24 hours.
4. 暂停、方案违背或提前终止临床研究，请及时上报本伦理委员会。
   Any suspension, project violation or early termination of the clinical research, should be reported to **ECCR** promptly.
5. 完成临床研究，须提交研究完成报告给本伦理委员会。
   Please submit a completion research report to **ECCR** after completion of the project.

温州医科大学附属第一医院临床研究伦理委员会
委员签到表

会议时间： 2021 年 06 月 24 日

会议地点： 新院 1-4A18 会议室

审查内容： ICA69 调节脓毒症性心功能障碍的分子机制研究 Molecular mechanism of ICA69 in regulating sepsis-induced cardiomyopathy in mice.

姓　名	性别	工作单位	专　业	职　称	伦理委员会职务	签到
吴高俊	男	温州医科大学附属第一医院	心血管内科	主任医师	主　任	/
黄晓颖	女	温州医科大学附属第一医院	呼吸内科	教授/主任医师	副主任	/
金　嵘	女	温州医科大学附属第一医院	流行病学	教授	委　员	/
张纯武	男	温州医科大学附属第一医院	伤骨科	主任中医师	委　员	/
陈咨苗	男	温州医科大学附属第一医院	内分泌科	副主任医师	委　员	/
陈必成	男	温州医科大学附属第一医院	实验外科学	主任技师	委　员	陈必成
蔡雪黎	女	温州医科大学附属第一医院	心内科	副教授/副主任医师	委　员	/
孙彩霞	女	温州医科大学附属第一医院	护　理	主任护师	委　员	/
陈　雷	男	温州医科大学附属第一医院	骨　科	教授/主任医师	委　员	/
郑祥武	男	温州医科大学附属第一医院	放射影像	教授/主任医师	委　员	/
陈永平	男	温州医科大学附属第一医院	感染内科	教授/主任医师	委　员	/
林观样	男	温州医科大学附属第一医院	药　学	主任药师	委　员	/
苏小芳	女	浙江震瓯律师事务所	法　律	律　师	委　员	/
胡建芬	女	退　休	统计学	高级统计师	委　员	/
方　耀	男	温州理工学院	伦理学	讲　师	委　员	方耀

伦理委员会声明：

★ 温州医科大学附属第一医院临床研究伦理委员会组成及工作程序遵循中国 GCP、ICH-GCP 及相关法律法规，其审查过程不受伦理委员会以外任何组织及个人影响。

★ 本伦理委员会各委员已签署保密协议，所有标准操作规程文件、机密信息、会议记录等及其副本的所有权均归伦理委员会。

地址：浙江省温州市瓯海区南白象温州医科大学附属第一医院新院区　　邮编：325000
联系电话：0577-55578027　　传真：0577-55578033　　E-mail:wyyyll01@163.com
