# Supplementary material for: ICA69 aggravates ferroptosis causing septic cardiac dysfunction via STING trafficking
Source: Cell Death Discov. 2022 Apr 9;8:187. doi: 10.1038/s41420-022-00957-y (PMC8994779; doi:10.1038/s41420-022-00957-y)
Supplement: Supplementary file 3 — Informed Consent Form [file 41420_2022_957_MOESM3_ESM.pdf]

ICA69 调节脓毒性心功能作用的分子机制研究的知情同意书

第一部分　知情部分

目前我们正开展一个关于 ICA69 调节脓毒症性心功能障碍的分子机制研究的研究项目。您将被邀请参加此项研究，该项目在温州医科大学附属第一医院开展。

在您决定是否参加这项研究之前，请尽可能仔细阅读以下内容。它可以帮助您了解该项研究以及为何要进行这项研究，研究的程序和期限，参加研究后可能给您带来的益处、风险和不适。如果您愿意，您也可以和您的亲属、朋友一起讨论，或者请医生给予解释，帮助您做出决定。

研究背景

脓毒症是一种全身炎症反应，伴随过度产生炎症细胞因子、氧化应激和多器官功能障碍。脓毒症引起的心肌病是脓毒症和脓毒症休克中普遍存在的并发症之一。胰岛细胞自身抗原 69 (ICA69)由 ICA1 基因编码，在组织和细胞中分布受限。此前的研究主要集中在ICA69在器官特异性自身免疫性疾病(如1型糖尿病(T1D)中的生理/病理作用。胸腺中 ICA69 表达缺失足以诱发多器官炎症。干扰素基因激因子 STING 由多个公认的跨膜区域组成，在静息状态下，主要作为同源二聚体锚定在 ER 膜内。STING 是 SIC 中不可缺少的一种分子，可调节炎症和免疫反应，并通过触发心肌细胞焦亡来参与脓毒症诱导的心脏损伤。这些结果提示 ICA69 可能参与 STING 依赖的先天免疫反应。铁死亡是一种活性氧(ROS)和铁依赖的调节细胞死亡形式，在器官损伤和癌症靶向治疗中发挥关键作用。最近的研究表明，高铁饮食或 Gpx4 缺失诱导铁死亡激活 sting 依赖的 DNA 传感器通路，最终导致巨噬细胞浸润和胰腺肿瘤发生。在细胞培养和动物模型中，STING 激活的遗传抑制可以防止铁死亡。STING 缺乏可缓解 LPS 处理小鼠肺部和巨噬细胞的炎症和氧化应激。脂多糖诱导的感染性心肌病小鼠心肌细胞铁死亡被激活，抑制铁死亡可减轻心脏炎症和功能障碍。然而，ICA69 和 STING 在铁死亡相关性心脏损伤中所起的作用尚无可用数据。

在细胞和分子水平上，炎症、凋亡和铁死亡被认为是脓毒症和 SIC 的重要病

理生理学现象。因此，能够选择性抑制上述过程的分子或基因对脓毒症和脂多糖引起的心功能障碍具有重要的治疗价值。

研究目的

本研究拟探讨 ICA69 调节脓毒症引起的心肌病作用的分子机制研究

哪些人可以被邀请参加本研究

根据脓毒症和脓毒症休克的第三个国际共识定义证实了患者的诊断。

哪些人不宜参加本研究

18 岁以下、孕妇、1 型糖尿病、发育不全或免疫抑制疾病(如 HIV)患者、接受免疫抑制治疗(化疗、长期使用类固醇、自身免疫性疾病治疗)或不明疾病的患者被排除在外

参加本研究项目的好处

为脓毒症患者的临床治疗提供一定的指导建议。

研究过程和方法：

提取脓毒症患者和健康对照者动脉血血浆 5ml，进行 qPCR 检测 ICA69, STING,GPX4 和 PTSG2 表达量。

参加本研究项目的风险：

本研究主要收集病人的动脉血进行检测，故可能有抽血后抽血点继续出血等风险。但发生率低，并将得到我们医护人员的密切观察，及时处理。如果你在研究期间出现任何不适，或病情发生新的变化，或任何意外情况，不管是否与研究相关，均应及时通知您的医生，您的医生将对此作出判断并给予适当的医疗处理。

隐私问题：

如果您决定参加本项研究，您参加试验及在试验中的个人资料均属保密。可以识别您身份的信息将不会透露给研究小组以外的成员，除非获得您的许可。所有的研究成员和研究申办方都被要求对您的身份保密。您的档案将保存在温州医科大学附属第一医院麻醉科，仅供研究人员查阅。为确保研究按照规定进行，必要时，政府管理部门或伦理审查委员会的成员按规定可以在研究单位查阅研究数据。

费用和补偿：

医生将尽全力预防伤害发生，如果发生了由于本研究带来的伤害，医生将会给予受试者积极的治疗，申办方将按照相关法律对与试验相关的损害提供治疗的费用及相应的经济赔偿。

对于您同时合并的其他疾病所需的治疗和检查，将不在免费的范围之内。

自由退出：

作为受试者，您可随时了解与本研究有关的信息数据和研究进展，自愿决定（继续）参加还是不（继续）参加。参加后，您可以选择在任何时候通知研究者要求退出研究，您的资料将不纳入研究结果，您的任何医疗待遇与权益不会因此而受到影响。如果继续参加研究，会对您造成严重的伤害，研究者也将会中止研究的进行。但在参加研究期间，请您提供有关自身病史和当身体状况的真实情况；告诉研究医生自己在本次研究期间所出现的任何不适；不得服用受限制的药物、食物等；告诉研究医生自己在最近是否曾参与其它研究，或目前正参与其它研究。如果因为您没有遵守研究计划，或者发生了与研究相关的损伤或者有任何其它原因，研究医师可以终止您继续参与本项研究。

如果您在参加本研究过程中有任何问题，请联系 王均炉 医师，联系电话： 13806689854 。

如果您有与受试者自身权益相关的问题，可以与临床研究伦理委员会联系，联系电话：0577-55578027。

第二部分同意部分

1．我已认真阅读该知情同意书，研究人员已经向我做了详尽说明并解答了我的关问题，我已充分知晓以上内容，同意参加研究。

受试者签名： 签名日期：

受试者法定代理人签名(必要时)： 签名日期：

2．我或我的研究人员已向该受试者充分解释和说明了本临床试验的目的、操作过程以及受试者参加该试验可能存在的风险和潜在的利益，并满意地回答了受试者的所有有关问题。

主要研究者或研究者指定的研究人员(对受试者进行告知者)签名：

签名日期：
