# Supplementary material for: ICA69 aggravates ferroptosis causing septic cardiac dysfunction via STING trafficking
Source: Cell Death Discov. 2022 Apr 9;8:187. doi: 10.1038/s41420-022-00957-y (PMC8994779; doi:10.1038/s41420-022-00957-y)
Supplement: Supplementary file 4 — Clinical Research and Ethical Review [file 41420_2022_957_MOESM4_ESM.pdf]

医学临床科研项目及伦理审查申请表

Application Form of Clinical Research and Ethical Review

<table>
<tr><td>方案名称
（中英对照）</td><td colspan="5">ICA69 调节脓毒症性心功能障碍的分子机制研究
Molecular mechanism of ICA69 in regulating sepsis-induced cardiomyopathy in mice</td></tr>
<tr><td>是否受基金资助及名称</td><td colspan="5">经费来源： 温州市科技局重大科技专项项目
项目名称： 基于电针脑缺血耐受效应构建脓毒症脑病防治策略及其机制研究</td></tr>
<tr><td>研究类别</td><td colspan="2">☑研究者发起
□合作项目</td><td>研究归类</td><td colspan="2">☑前瞻临床研究 ☑课题申报
□回顾性研究 ☑文章投稿</td></tr>
<tr><td>组长单位</td><td></td><td>主中心负责人</td><td></td><td>已有的相关伦理批件</td><td>□有 □无</td></tr>
<tr><td>我院项目负责人</td><td>王均炉</td><td>职称</td><td>主任医师</td><td>所在科室</td><td>麻醉科</td></tr>
<tr><td>研究期限</td><td>2021.06-2022.06</td><td>经费</td><td>10 万</td><td>例数</td><td>150</td></tr>
</table>

研究方法涉及内容：（在适当项目内打勾）

☑观察性研究 □有临床干预 □药物干预

☑数据采集（□门诊、☑住院、□医技、□其它____ ） □有创性干预 □其它_______

□问卷式调查 ☑统计分析 □流行病学调查

样本采集：（在适当项目内打勾）

□有新增有创性样本采集 □使用丢弃或保存的组织样品 ☑使用血、尿样本

□涉及遗传物质的运输： □国内 □国外 □其他__________

<table>
<tr><td rowspan="2">回顾性研究填写</td><td>既往何时的病案数据/标本</td><td>年 月 日—— 年 月 日</td></tr>
<tr><td>进行后续随访</td><td>□是（需提供知情同意书） □否</td></tr>
<tr><td colspan="2">涉及的药品、耗材、器械</td><td>名称： 院内已有：□是 □否</td></tr>
</table>

<table>
<tr><td>安全性</td><td>是否增加有创性检测或干预： □是 □否
是否增加超说明书用药：□是 □否
指南中是否有此诊疗项： □是 □否
是否涉及干细胞、基因治疗等重大创新技术:□是□否
项目负责人确认签字： 日期：</td><td>科室主任填写并签字： 日期：
该项目的临床意义：□大 □中 □小
潜在风险是否增加：□是 □否
项目的可行性：□难以开展 □具备条件
□同意开展 □不同意开展</td></tr>
</table>

如果涉及药物：

1、是否存在超说明书使用：□是 □否 2、目前药物是否在药学部管理：□是 □否

药学部意见：____

药学部主任签字： 日期：

学科规划与科技处/临床研究中心审核意见： 风险点：1. ______________

科研工作获益：□大 □中 □小 2. ______________

项目潜在风险：□无 □ 高 □中 □低 3. ______________

意见：____

学科规划与科技处处长/临床研究中心主任签字： 日期：

____**项目（必填）**

送审项目资料清单

	编号	资料名称	资料完整		版本号（例：2018V2.0 版）	版本日期
			有	无		
伦理备案	1	医学临床科研项目及伦理审查申请表*	☐	☐		
	2	临床研究方案*	☐	☐		
	3	受试者知情同意书/免除知情同意申请*	☐	☐		
	4	研究者团队成员目录（职责）*	☐	☐		
	5	主要研究者、团队成员简历及 GCP 证书*	☐	☐		
	6	研究者责任声明*	☐	☐		
	7	CRF/临床观察表样板*	☐	☐		
	8	廉政承诺	☐	☐		
	9	产品说明书/指南（如果有指南依据，请打印相关一页）	☐	☐		
	10	中心伦理批件	☐	☐		
	11	超说明书用药备案材料	☐	☐		
	12	外来研究药品/耗材/设备管理备案材料	☐	☐		
	13	保险合同	☐	☐		
	14	研究者手册	☐	☐		
	15	《药物临床研究批件》/注册批件	☐	☐		
	16	企业资质：营业执照/生产许可证/GMP 证书	☐	☐		
	17	第三方机构资质及委托书	☐	☐		
	18	受试者筛选与入选登记表样张	☐	☐		
	19	受试者鉴认代码表样张	☐	☐		
	20	其他备案所需材料	☐	☐		
科教处/临床研究中心备案	21	科研项目申请书*	☐	☐		
	22	立项文件（合作项目/基金会项目提供）*	☐	☐		
	23	任务书/研究协议/合同*	☐	☐		
	24	经费预算说明（有经费资助提供）*	☐	☐		

备注： 1-7 及 21-24 带*为必备项，其他资料如有适用也需提供（回顾性研究提供材料 1-7 即可），材料纸质版一式一份双面打印交至科教处，经审核后由科教处或临床研究中心递交至伦理办公室，电子版发送至（wyyyclinical@126.com）。本文档打印前请删除批注！

送审人签名：　　　　　　联系方式：　　　　　　送审日期：
