# Supplementary material for: ICA69 aggravates ferroptosis causing septic cardiac dysfunction via STING trafficking
Source: Cell Death Discov. 2022 Apr 9;8:187. doi: 10.1038/s41420-022-00957-y (PMC8994779; doi:10.1038/s41420-022-00957-y)
Supplement: Supplementary file 5 — Ethical Inspection ID wydw2019-0559 [file 41420_2022_957_MOESM5_ESM.pdf]

温州医科大学实验动物中心动物实验伦理审查表

The Tab of Animal Experimental Ethical Inspection of Laboratory Animal Centre, Wenzhou Medical University

批准编号(ID Number)：

申请人填写的相关信息 (Related information filled by applicant)

申请单位(Name of organization)：温州医科大学附属第一医院 The First Affiliated Hospital Of *Wenzhou Medical University*

申请人学历 (Education of applicant)：本科 bachelor	技术职称 (Professional title)：主任医师 archiater

实验名称(Experiment title)：基于 PICK1/PP2A 复合物调控肺内皮细胞钙内流介导的电针抗脓毒症肺损伤的机制研究 （Study on the mechanism of electroacupuncture in the treatment of septic lung injury based on the PICK1/PP2A complex regulating intracellular calcium influx in pulmonary endothelial cells）

项目来源(Project sources)：省基础公益研究计划（Provincial basic public welfare research plan）

实验目的(Aim of experiment)：研究电针治疗脓毒症肺损伤作用机制 To study the mechanism of electroacupuncture in the treatment of sepsis lung injury

实验动物使用许可证号(Number of the using of Laboratory Animal)：SYXK（浙）2015-0009

拟进动物情况

动物来源(Source of animal)：温州医科大学实验动物中心 Laboratory Animal Centre, Wenzhou Medical University

品种品系(Species or strain)：C57BL/6 等级(Grade)： SPF 规格(Specifications)：18-22g

数量(Number)： 400 只 (♀ 只；♂ 400 只)	申请日期(Application date)： 2020 年 5 月 18 日
进驻日期(Entering date)： 2020 年 5 月 31 日	结束日期(Ending date)： 2022 年 12 月 31 日

实验要点，包括实验方法、观测指标、实验结束后处死动物的方法等：
(Outline of experiments, experimental methods, observational index, executing animal method, et. al)：

该研究本着实验动物福利和伦理的原则，本实验项目优化设计方案，严格计划动物需要数量，计划需要 400 只 C57BL/6 小鼠。采用 $PICK1^{\Delta EC}$、$ICA69^{\Delta EC}$ 以及野生型（WT）小鼠,构建脓毒症急性肺损伤模型,给予电针处理,行肺泡灌洗液、电镜、H&E 染色、Western Blo、流式细胞荧光分选技术，检测内皮细胞相关蛋白 ICAM-1、VCAM-1 蛋白、Ca2+浓度、NFAT 细胞核转移情况以及荧光素酶检测 NFAT 活性。实验结束后，经打包，最后统一焚烧。

In this study, based on the principles of laboratory animal welfare and ethics, the experimental project was designed to optimize the number of animals, and 400 C57BL/6 mice were planned.Using PICK1 Δ EC and wild type mice (WT), sepsis acute lung injury model was constructed.Give electroacupuncture treatment,Alveolar lavage solution, electron microscopy, H&E staining, WB and FACS were used to detect the ICAM-1, VCAM-1, Ca2+ concentration, NFAT nuclear transfer, and luciferase activity of endothelial cell related proteins.After the experiments, all mice were burned.

申请人(Name of applicant)：王均炉　　联系电话 (Telephone)：13806689854

项目负责人 (Name of Project director)： 王均炉　　联系电话 (Telephone)：13806689854

声明(Statement)：

我将自觉遵守实验动物福利伦理原则，随时接受委员会的监督与检查，如违反规定，自愿接受处罚。

(I will conscientiously abide by the ethical principles of animal welfare, accept the supervision and inspection of the committee at any time, and voluntarily accept the punishment if any infringement.)

项目负责人签名(Signature of Project director)：

项目执行人签章(Signature of Project implementation)：

**审查依据** (Inspection contents)	1. 该项目是否必须用实验动物进行实验，即能否用计算机模拟、细胞培养等非生命方法替代动物或用低等动物替代高等动物进行实验？<br>(Does laboratory animal must be used in the project? Could other methods such as computer simulation, cell cultivation or using the low-grade animal instead of the high-grade animal?)<br><br>2. 表中所填申请人资格和所用动物的品种品系、质量等级、规格是否合适，能否通过改良设计方案或用高质量的动物来减少所用动物的数量？<br>(Are the qualification of applicant，species or strain, grade and specifications of animals suitable? Could the quantity of animals be reduced by improving the study design or using high quality animals?)<br><br>3. 能否通过改进实验方法、调整实验观测指标、改良处死动物的方法，来优化实验方案、善待动物？<br>(Could the study design and animal treatment be refined by ameliorating experimental method, adjusting observational index, executing animal method?)
**审查结果** (是否同意申请人的实验方案) (Results of inspection)	实验动物管理和伦理委员会意见 (Attitude of the Animal Management and Ethics Committee)：　同意 (Agree) ☐　不同意 (Disagree) ☐　修改后同意（Agree after revised） ☐

伦理委员会主任委员签名(Signature of Ethics Committee Director)：

温州医科大学实验动物伦理委员会
温州医科大学实验动物中心
（代章）
(Laboratory Animal Ethics Committee of Wenzhou Medical University & Laboratory Animal Centre of Wenzhou Medical University)
(Stamp)
年　　月　　日

填表说明：

1、全部填写完后，如果表格变成 3 页，调整一下，并删除此页的填表说明。所填内容需要中英文对照。

2、实验动物使用许可证号填写如下：

大鼠、小鼠等实验动物用：SYXK（浙）2015-0009

兔子、豚鼠、犬等实验动物用：SYXK（浙）2019-0009

3、申请人可以填写为硕士研究生等经办人，项目负责人应该填写为导师、项目申请负责人等，联系电话填手机全号。

4、动物数量一定要填写，要严格控制数量。

5、伦理审查表，要求在实验过程中要善待动物，饲养、操作规范，采用安乐死处死，不能直接断头、颈椎脱臼等残忍方式处死动物，必须麻醉，尸体打包，统一无害化处理，否则难以通过审查。

6、本审查表仅用于 2021 年度省基础公益研究计划项目申请。温州医科大学具有实验动物使用许可证的动物为：大鼠、小鼠、兔子、豚鼠、犬，因此除此之外的动物不予受理。

7、实验动物伦理委员会审查需提供的附件材料：（1）实验动物伦理委员会审查表（2）项目实验方案
